# A disrupted FOXP3 transcriptional signature underpins systemic regulatory T cell insufficiency in early pregnancy failure

**DOI:** 10.1016/j.isci.2024.108994

**Published:** 2024-01-23

**Authors:** Lachlan M. Moldenhauer, Kerrie L. Foyle, Jasmine J. Wilson, Ying Y. Wong, David J. Sharkey, Ella S. Green, Simon C. Barry, M. Louise Hull, Sarah A. Robertson

**Affiliations:** 1 Robinson Research Institute and School of Biomedicine, The University of Adelaide, Adelaide, SA, Australia; 2 Robinson Research Institute and Adelaide Medical School, The University of Adelaide, Adelaide, SA, Australia

**Keywords:** Health sciences, Pregnancy, Immunology

## Abstract

Regulatory T (Treg) cell defects are implicated in disorders of embryo implantation and placental development, but the origins of Treg cell dysfunction are unknown. Here, we comprehensively analyzed the phenotypes and transcriptional profile of peripheral blood Treg cells in individuals with early pregnancy failure (EPF). Compared to fertile subjects, EPF subjects had 32% fewer total Treg cells and 54% fewer CD45RA^+^CCR7^+^ naive Treg cells among CD4^+^ T cells, an altered Treg cell phenotype with reduced transcription factor FOXP3 and suppressive marker CTLA4 expression, and lower Treg:Th1 and Treg:Th17 ratios. RNA sequencing demonstrated an aberrant gene expression profile, with upregulation of pro-inflammatory genes including *CSF2*, *IL4*, *IL17A*, *IL21*, and *IFNG* in EPF Treg cells. *In silico* analysis revealed 25% of the Treg cell dysregulated genes are targets of FOXP3. We conclude that EPF is associated with systemic Treg cell defects arising due to disrupted FOXP3 transcriptional control and loss of lineage fidelity.

## Highlights

• Women with early pregnancy failure (EPF) have fewer peripheral blood CD4^+^ Treg cells
• The phenotype of Treg cells in EPF is skewed toward reduced suppressive competence
• Transcriptional analysis shows loss of FOXP3 regulation and a pro-inflammatory shift
• EPF is associated with systemic Treg cell defects similar to autoimmune conditions

## Graphical abstract

**Figure:**
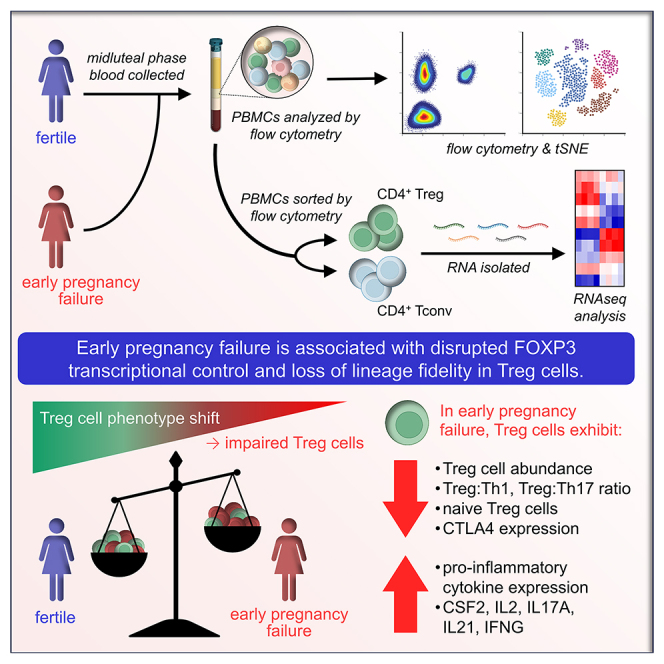

## Introduction

Maternal-fetal immune tolerance is critical for embryo implantation and development of a robust placenta to support healthy fetal development and on-time birth. Impaired immune tolerance is implicated in disorders of uterine receptivity to implantation that give rise to early pregnancy failure (EPF), manifesting as recurrent implantation failure and/or recurrent pregnancy loss. These conditions are common and frustratingly intractable, with limited treatment interventions.^1^^,^^2^^,^^3^ Recurrent pregnancy loss (also termed “recurrent miscarriage”), defined as two or more successive spontaneous pregnancy losses, affects 1%–3% of women.^2^^,^^4^ Genetic abnormalities of the embryo or parents and maternal endocrine disorders are identified risk factors, but the cause is unknown in approximately 50% of cases.^1^^,^^5^ Recurrent implantation failure is the repeated failure of normal embryos to implant successfully and occurs in an estimated 10% of women seeking reproductive medicine treatment.^3^^,^^6^ There is accumulating evidence pointing to immune aberrations as a causal factor in many women who experience recurrent pregnancy loss^1^^,^^7^ and recurrent implantation failure,^8^ but defining the precise nature of immune lesions remains a challenge and limits clinical progress.^9^

Key mediators of maternal-fetal tolerance are CD4^+^FOXP3^+^ regulatory T (Treg) cells, which accumulate in the decidualized lining of the uterus where they act during embryo implantation to inhibit excessive inflammation and suppress development of immunity to paternally inherited transplantation antigens on the embryo.^10^^,^^11^ Recruited Treg cells include cells generated in the periphery as well as locally in the gestational tissues, where differentiation of CD4^+^ T cells into Treg cells is induced by human leukocyte antigen-G (HLA-G) expressed on extravillous trophoblast cells invading the decidua.^12^^,^^13^ These activated Treg cells interact with other immune cells to facilitate remodeling of the uterine spiral arteries necessary for robust placental development.^14^^,^^15^ Their abundance and functional competence are controlled by immune-regulatory cytokines, hormones, and alloantigens of paternal origin.^11^ Mouse studies show that during each reproductive cycle, the Treg cell pool expands prior to receptivity to embryo implantation, and this involves recruitment from the peripheral blood of naive Treg cells that are primed in reproductive tract tissues by sex hormones and seminal fluid components.^15^^,^^16^^,^^17^^,^^18^ A sufficient supply of naive Treg cells of predominantly thymic origin is required to resource the uterus,^19^^,^^20^ particularly for establishing first pregnancy, in which induced Foxp3 expression in naive T cells and proliferation of recruited Foxp3^+^ thymic Treg cells are crucial to support acquisition of protective regulatory memory.^21^ Similar mechanisms to recruit and expand uterine Treg cells are implicated in women.^22^^,^^23^^,^^24^

Several studies show that Treg cells are reduced in number and altered in phenotype in the uterine decidua of women with recurrent implantation failure^25^ and recurrent pregnancy loss.^26^^,^^27^^,^^28^^,^^29^ Similar changes are implicated in a range of pregnancy complications to which altered placental development contribute, including preeclampsia^30^ and preterm labor.^31^^,^^32^ Treg cell defects are likely to arise systemically, as a similar reduction in Treg cells as a fraction of CD4^+^ T cells occurs in the peripheral blood,^7^^,^^33^^,^^34^^,^^35^ with reduced Treg cell suppressive capacity^36^^,^^37^ and increased pro-inflammatory Th17 cells.^38^^,^^39^^,^^40^^,^^41^ Elevated interferon gamma (IFNG) and reduced IL10 synthesis in peripheral blood T cells indicate a systemic pro-inflammatory disposition in these conditions.^42^^,^^43^ A causal contribution of Treg cells to embryo implantation and placentation defects is indicated by experimental depletion of Treg cells in mice. Depending on the timing and degree of Treg cell deficiency, both implantation failure and later fetal loss can ensue.^15^^,^^44^^,^^45^

A lack of clarity on the immune etiologies that cause EPF and the particular significance of T cells and Treg cells have hampered development of diagnostics and therapeutic interventions for recurrent pregnancy loss and recurrent implantation failure.^9^^,^^46^ In previous studies that have quantified Treg cells in women with EPF, phenotyping panels are often limited in scope and there is no consensus understanding of the ontogeny of Treg cell deficiency.^7^^,^^47^ Not all individuals with recurrent pregnancy loss show altered Treg cells, and not all studies report consistent findings (see meta-analysis by Keller et al^7^). This underscores the need for appropriately powered studies with sufficiently detailed phenotype analysis to enable confident ascertainment of the role of Treg cells in pregnancy loss pathophysiology.

Despite emerging understanding of Treg cell phenotypic instability arising from altered gene regulatory networks as a cause of several autoimmune and inflammatory conditions,^48^ this has not been evaluated in the reproductive setting. A recent study found altered gene expression in endometrial Treg cells of women with recurrent pregnancy loss but did not determine whether Treg cell changes reflect systemic defects detectable in the peripheral blood.^29^ We therefore hypothesized that systemic defects in Treg cell stability are associated with Treg cell defects in EPF. To evaluate this, we undertook a detailed immunophenotyping analysis of Treg, CD4^+^ Tconv, and CD8^+^ T cell populations in women with EPF and women with proven fertility, using a multi-parameter flow cytometry panel to investigate markers of suppressive capacity, proliferation state, and memory and naive subsets. We then prepared pure populations of Treg cells and conventional CD4^+^ T cells (Tconv cells) by fluorescence-activated cell sorting (FACS) and profiled their gene expression by RNA sequencing (RNA-seq) and *in silico* bioinformatics analysis. Our data reveal new insight on previously undetected Treg cell molecular aberrations that indicate systemic Treg cell defects in this common condition.

## Results

### Treg cells are fewer and have an altered phenotype in EPF

**Figure 1:**
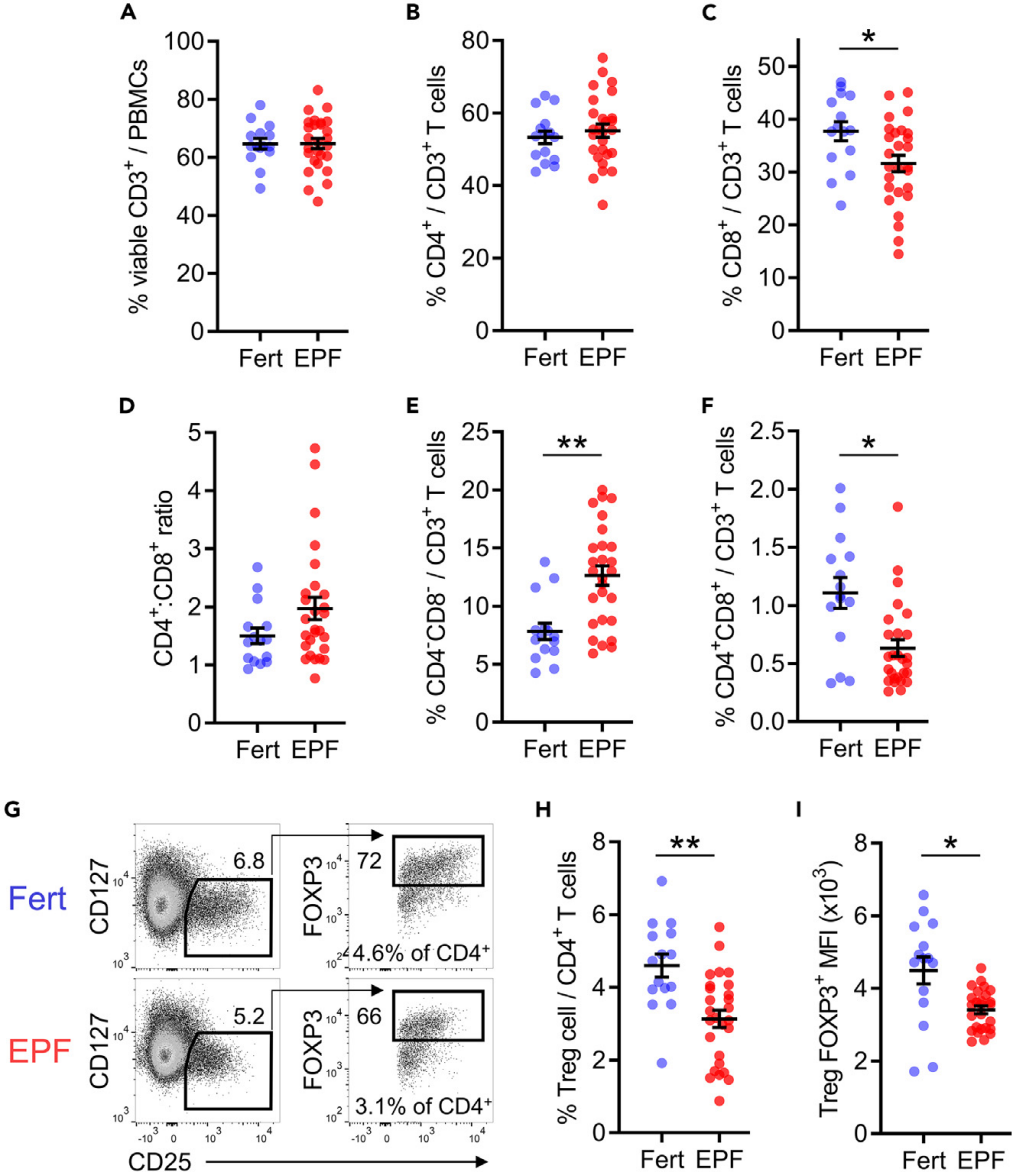
Treg cells are less abundant with reduced FOXP3 in early pregnancy failure Peripheral blood lymphocytes were assessed by flow cytometry in proven fertile (Fert, n = 15) and early pregnancy failure subjects (EPF, n = 27). The population of viable CD3^+^ T cells (A) was assessed for CD4 and CD8 expression to define the percentage of CD4^+^CD8^−^ (B) and CD8^+^CD4^−^ (C) T cells, and the CD4^+^:CD8^+^ ratio was calculated (D). The proportion of double-negative CD4^−^CD8^−^ (E) and double-positive CD4^+^CD8^+^ (F). Within the CD3^+^CD4^+^ population, FOXP3^+^ CD25^+^CD127^-/lo^ Treg cells were identified (G) and quantified (H), then within the Treg cell pool the MFI of FOXP3 (I) was measured. Symbols indicate individual study participants, and the mean ± SEM of each group are shown. The effect of fertility status was analyzed by t test with FDR correction to account for multiple comparisons. ∗p < 0.05, ∗∗p < 0.01.

To evaluate the relationship between EPF and peripheral blood T cells, we utilized 15-color flow cytometry to analyze peripheral blood mononuclear cells (PBMCs) collected at the mid-luteal phase of the menstrual cycle from subjects with previous EPF (n = 27) or proven fertility (n = 15). EPF subjects had clinical features of recurrent pregnancy loss, recurrent implantation failure, or a combination of both (see STAR Methods section for more information, and Tables S1 and S2 for clinical details). There was no difference in EPF subjects in the proportion of CD4^+^ T cells within the CD3^+^ T cell population (Figures 1A and 1B). CD8^+^ T cells were 16% fewer among CD3^+^ T cells in the EPF group compared to fertile subjects (Figure 1C, p = 0.028), but the shift in CD4^+^:CD8^+^ ratio did not reach statistical significance (Figure 1D, p = 0.087). EPF subjects had a 61% increase in double-negative (CD4^−^CD8^−^) T cells (Figure 1E, p = 0.001) and a 43% decrease in double-positive (CD4^+^CD8^+^) T cells (Figure 1F, p = 0.016) compared to fertile subjects.

There were substantial differences in Treg cell numbers in EPF subjects. Within the CD4^+^ T cells, the proportion of FOXP3^+^CD25^+^CD127^-/lo^ Treg cells was 32% lower in the EPF group (Figures 1G and 1H, p = 0.005). The mean fluorescent intensity (MFI) of the signature Treg cell transcription factor FOXP3 in FOXP3^+^ Treg cells was 20% less in EPF than in fertile subjects (Figure 1I, p = 0.027).

**Figure 2:**
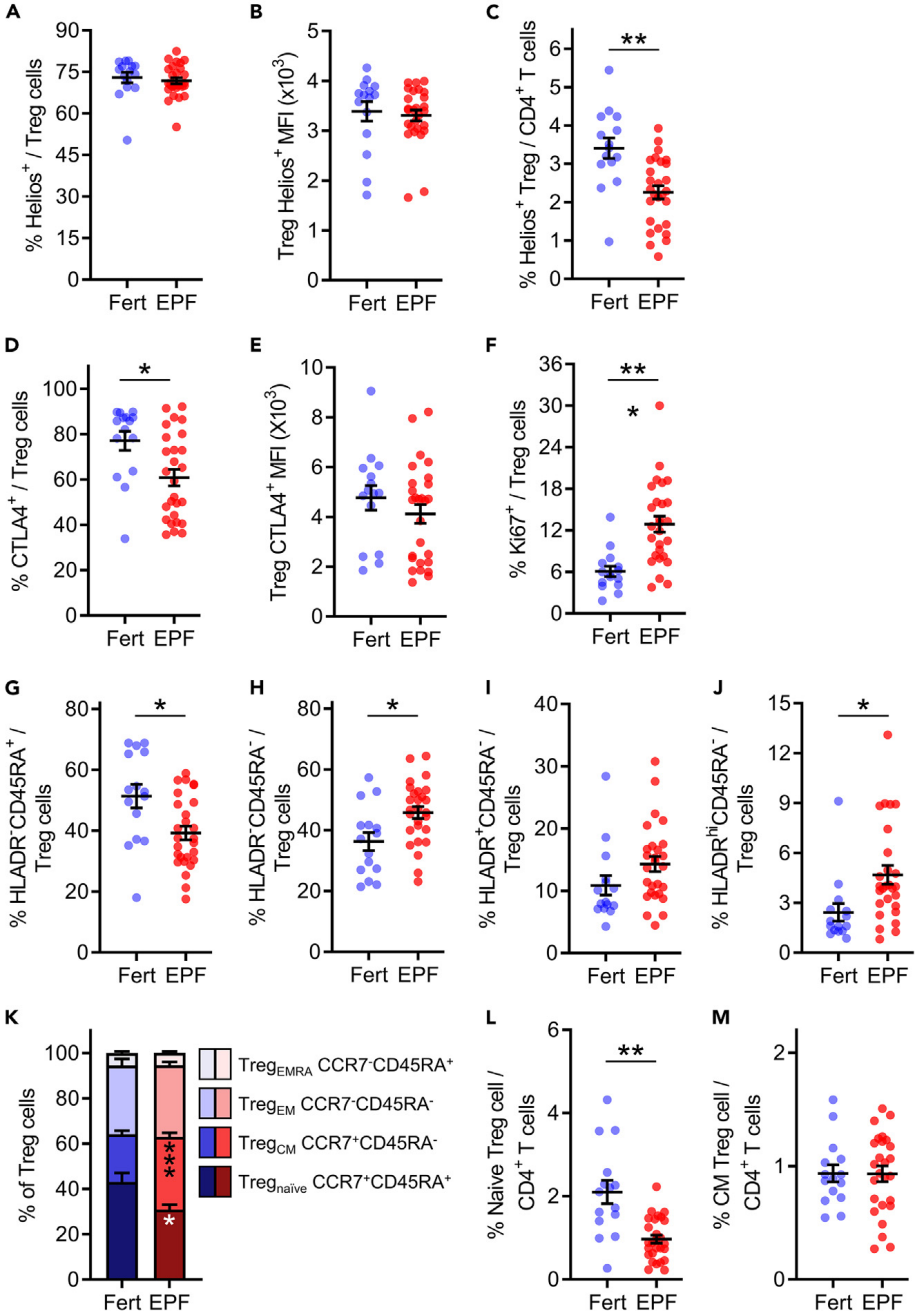
Treg cells have an altered phenotype indicating reduced functional fitness in early pregnancy failure Peripheral blood lymphocytes were assessed by flow cytometry and CD25^+^FOXP3^+^CD127^-/lo^ Treg cells within the CD3^+^CD4^+^ pool were analyzed in proven fertile (Fert, n = 15) and early pregnancy failure subjects (EPF, n = 27) (A). Within the Treg cell pool the percentage of Helios^+^ cells (A) and the MFI of Helios within Helios^+^ Treg cells (B) was measured. Helios^+^ Treg cells as a proportion of CD4^+^ T cells (C). The proportion of Treg cells expressing suppressive marker CTLA4 (D) and the MFI of CTLA4 within CTLA4^+^ Treg cells (E). The proportion of Treg cells expressing proliferative marker Ki67 (F). The proportion of Treg cells according to degree of suppressive capacity indicated by HLADR^−^CD45RA^+^ (G), HLADR^−^CD45RA^−^ (H), HLADR^+^CD45RA^−^ (I) and HLADR^hi^CD45RA^−^ (J). Treg memory and naive phenotypes according to CCR7 and CD45RA expression (K) identifying naive (Treg_naive_), central memory (Treg_CM_), effector memory (Treg_EM_) and terminally differentiated effector memory cells expressing CD45RA (Treg_EMRA_) Treg cells. The proportion of Treg_naive_ and Treg_CM_ within the CD4^+^ population are shown in (L) and (M), respectively. For (A-J, L, M), symbols indicate individual study participants, and the mean ± SEM of each group are shown. For (K), the subtypes of memory and naive Treg cells are shown as a stacked bar graph with the mean ± SEM of each subgroup indicated. The effect of fertility status was analyzed by t test with FDR correction to account for multiple comparisons. ∗p < 0.05, ∗∗p < 0.01, ∗∗∗p < 0.001.

The phenotype of Treg cells in EPF subjects was also different. Helios, a marker of FOXP3 expression stability and Treg cell suppressive capacity,^49^ was unchanged in Treg cells of EPF subjects in terms of proportion of Treg cells, or MFI (Figures 2A and 2B), although the proportion of Helios^+^ Treg cells within the whole CD4^+^ T cell population was reduced by 34% (Figure 2C, p = 0.006). The proportion of Treg cells expressing CTLA4, a marker of suppressive capacity, was reduced by 21% in the EPF group (Figure 2D, p = 0.019). There was no difference in CTLA4 MFI within CTLA4^+^ Treg cells (Figure 2E). EPF Treg cells expressed higher levels of the proliferation marker Ki67, with a 2.1-fold increase in Ki67^+^ Treg cells compared to Treg cells of fertile subjects (Figure 2F, p < 0.001). Altered phenotype parameters showed relationships with Treg cell frequency (Figure S1); for example, elevated Ki67 was particularly evident in EPF subjects with low Treg cells (Figure S1A).

Human leukocyte antigen-DR (HLADR) and CD45RA are markers of the maturity and suppressive capacity of Treg cells. CD45RA^+^ cells are considered weak suppressors, while HLADR^+^ Treg are strong suppressors, and HLADR^hi^CD45RA^−^ Treg cells have the maximum suppressive capacity.^50^^,^^51^ HLADR^−^CD45RA^+^ Treg cells were 24% lower in the EPF group (Figure 2G, p = 0.028), while the more suppressive double-negative HLADR^−^CD45RA^−^ Treg cells were elevated by 1.3-fold (Figure 2H, p = 0.027). There was no change in the proportion of highly suppressive HLADR^+^CD45RA^−^ Treg cells (Figure 2I), while the most suppressive HLADR^hi^CD45RA^−^ Treg cell subset were elevated in EPF by 1.9-fold (Figure 2J, p = 0.019), and this was most notable in EPF subjects with low Treg cells (Figure S1C).

Similar effects on Treg cell number and phenotype were seen when subgroups of EPF subjects that met strict diagnostic criteria for recurrent pregnancy loss and recurrent implantation failure were considered. Treg cells were reduced by 34% and 31% among CD4^+^ T cells in women with recurrent pregnancy loss and recurrent implantation failure, respectively (Figure S2A both p < 0.01). Elevated Ki67 (Figure S2D) and elevated HLADR (Figure S2F, both p < 0.05) were detected in both subgroups, while reduced CTLA4 was evident in the recurrent implantation failure but not the recurrent pregnancy loss subgroup (Figure S2C).

To assess the memory and naive phenotypes of Treg cells, CCR7 and CD45RA were evaluated. Treg cells can be defined as naive (Treg_naive_, CCR7^+^CD45RA^+^), central memory (Treg_CM_, CCR7^+^CD45RA^−^), effector memory (Treg_EM_, CCR7^−^CD45RA^−^), or terminally differentiated effector memory expressing CD45RA (Treg_EMRA,_ CCR7^−^CD45RA^+^).^52^ Within the total Treg cell pool of EPF subjects there was a 28% decrease in the proportion of Treg_naive_ cells and a 34% increase in the proportion of Treg_CM_ cells, compared to fertile subjects (Figure 2K, both p < 0.05), while Treg_EM_ and Treg_EMRA_ were comparable (Figure 2K). Because of the substantial reduction in Treg cells among CD4^+^ cells, this corresponds to a 54% reduction in Treg_naive_ cells, while Treg_CM_ were unchanged as a fraction of CD4^+^ cells (Figures 2L and 2M). Comparable reductions in Treg_naive_ as a fraction of CD4^+^ T cells were also evident in subject subgroups meeting diagnostic criteria for recurrent pregnancy loss (52%, p = 0.019) and recurrent implantation failure (55%, p = 0.016) (Figure S2I). When Ki67 was evaluated as a function of memory phenotype, Treg_naive_ and Treg_EMRA_ were substantially more proliferative in EPF than in fertile subjects, while Treg_CM_ and Treg_EM_ were similar (Figure S3).

Collectively, the flow cytometry analysis revealed reduced abundance of CD4^+^ Treg cells in EPF subjects, with lower FOXP3 and CTLA4 expression, but retention of a HLADR^hi^ subset. Elevated Ki67 expression indicated a greater proportion of Treg cells were proliferating in EPF subjects, and analysis of memory phenotypes showed the deficit was associated with fewer naive Treg cells among the Treg cell pool.

### CD4^+^ Tconv cells have a pro-inflammatory shift in EPF

**Figure 3:**
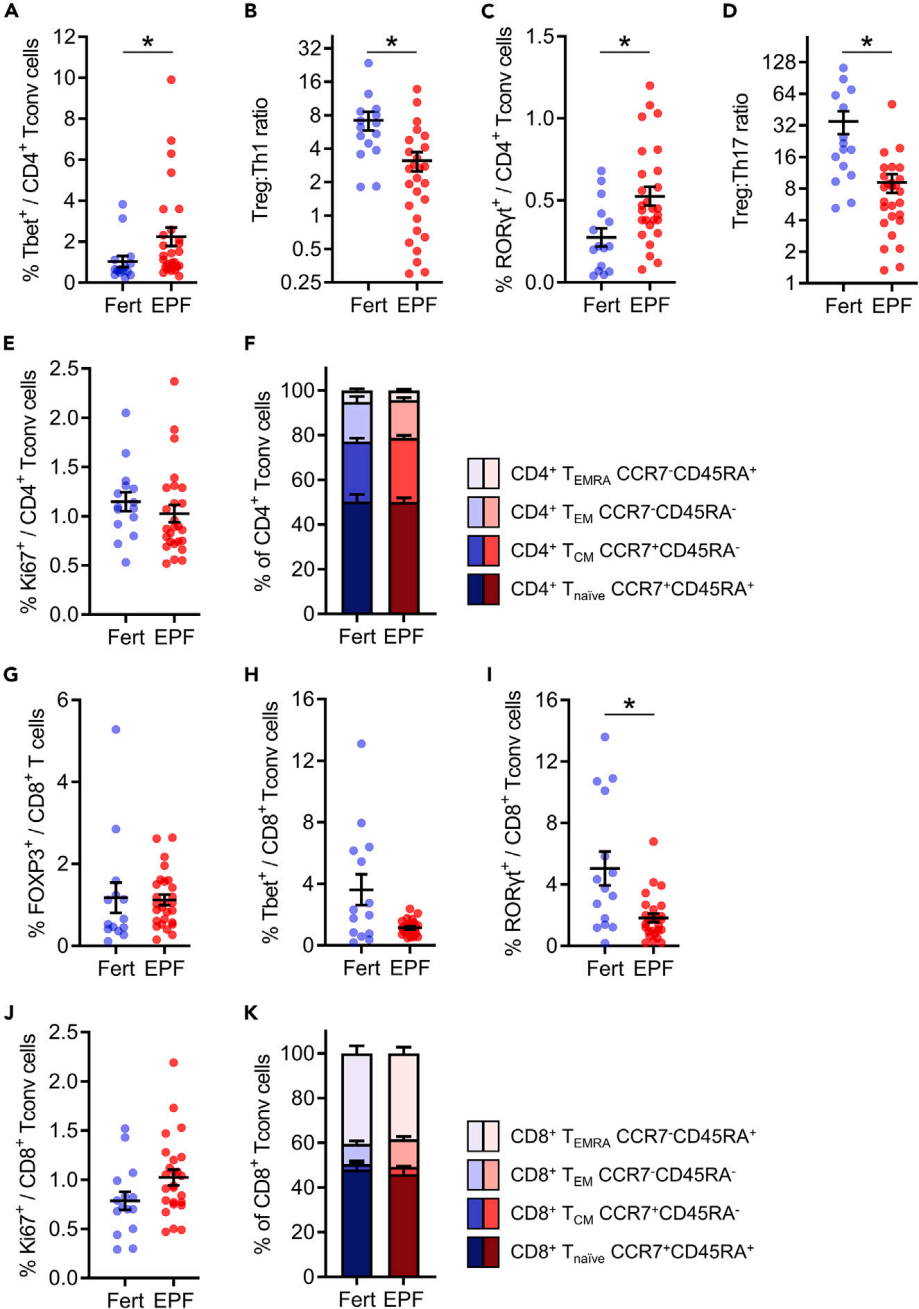
CD4^+^ and CD8^+^ T cells exhibit altered phenotypes in early pregnancy failure Peripheral blood CD4^+^ (A–F) and CD8^+^ (G–K) T cells were assessed by flow cytometry in proven fertile (Fert, n = 15) and early pregnancy failure subjects (EPF, n = 27). CD4^+^ T conventional (Tconv) cells were defined as CD3^+^CD4^+^ and not FOXP3^+^CD25^+^CD127^-/lo^ and CD8^+^ T cells were defined as FOXP3^+^CD8^+^ Treg cells (G) or FOXP3^−^CD8^+^ Tconv cells (H–K). Tbet expression was measured in CD4^+^ Tconv (A) and CD8^+^ Tconv cells (H) and the ratio of CD4^+^ Treg cells to CD4^+^ Th1 cells (Treg:Th1, B) was calculated. RORγt expression was measured in CD4^+^ Tconv (C) and CD8^+^ Tconv cells (I), and the ratio of CD4^+^ Treg cells to CD4^+^ Th17 cells (Treg:Th17, D) was calculated. The percentage of proliferating CD4^+^ Tconv (E) and CD8^+^ Tconv (J) cells was evaluated by Ki67 expression. CD4^+^ Tconv (F) and CD8^+^ Tconv (K) memory and naive phenotypes were measured by assessing CCR7 and CD45RA expression to define; naive (T_naive_), central memory (T_CM_), effector memory (T_EM_) and effector memory expressing CD45RA (T_EMRA_) Treg cells. For (A–E) and (G–J), symbols indicate individual study participants, and the mean ± SEM of each group are shown. For (F) and (K), memory and naive phenotypes are shown as a stacked bar graph with the mean ± SEM of each subgroup. The effect of fertility status was analyzed by t test with FDR correction to account for multiple comparisons. ∗p < 0.05.

We then assessed the activation phenotype of the CD4^+^ Tconv cells of subjects with EPF or proven fertility, by measuring expression of Tbet, the Th1 cell-defining transcription factor, and RORγt, the Th17 cell-defining transcription factor. A greater proportion of CD4^+^ Tconv cells expressed Tbet in EPF patients (Figure 3A, p = 0.048). Because of fewer Treg cells and more Th1 cells, the ratio of Treg:Th1 cells was decreased in EPF subjects by 57% (Figure 3B, p = 0.023). The percentage of Tconv cells expressing RORγt was elevated by 1.9-fold in EPF subjects (Figure 3C, p = 0.013), causing a 74% decline in the Treg:Th17 cell ratio (Figure 3D, p < 0.027). These parameters indicate a pro-inflammatory shift in CD4^+^ Tconv cells in EPF subjects. The proportion of CD4^+^ Tconv cells expressing Ki67 was similar in fertile and EPF subjects (Figure 3E), and their memory phenotype distribution was also comparable (Figure 3F). A reduced Treg:Th1 ratio was evident in both recurrent pregnancy loss and recurrent implantation failure (both p < 0.05, Figure S2K), and a reduced Treg:Th17 ratio was also evident in both groups (both p < 0.03, Figure S2L).

### CD8^+^ Tconv cells have an altered phenotype in EPF

When CD8^+^ T cells were analyzed, the proportion expressing FOXP3 (CD8^+^FOXP3^+^ Treg cells) was not changed in EPF subjects (Figure 3G). Within Tconv CD8^+^ T cells (CD8^+^FOXP3^-^), the percentage of pro-inflammatory CD8^+^ T cells expressing the CD8^+^ T cytotoxic-1 (Tc1) cell transcription factor Tbet was unchanged (Figure 3H), although the proportion expressing the CD8^+^ T cytotoxic-17 (Tc17) cell transcription factor RORγt was reduced by 64% (Figure 3I, p < 0.029). There was a trend toward elevated proliferation in Tconv CD8^+^ T cells as measured by Ki67 expression (Figure 3J, p = 0.068), but no change in their memory phenotype distribution (Figure 3K), in subjects with EPF compared to fertile subjects.

### tSNE analysis confirms CD3^+^ T cell changes in EPF

**Figure 4:**
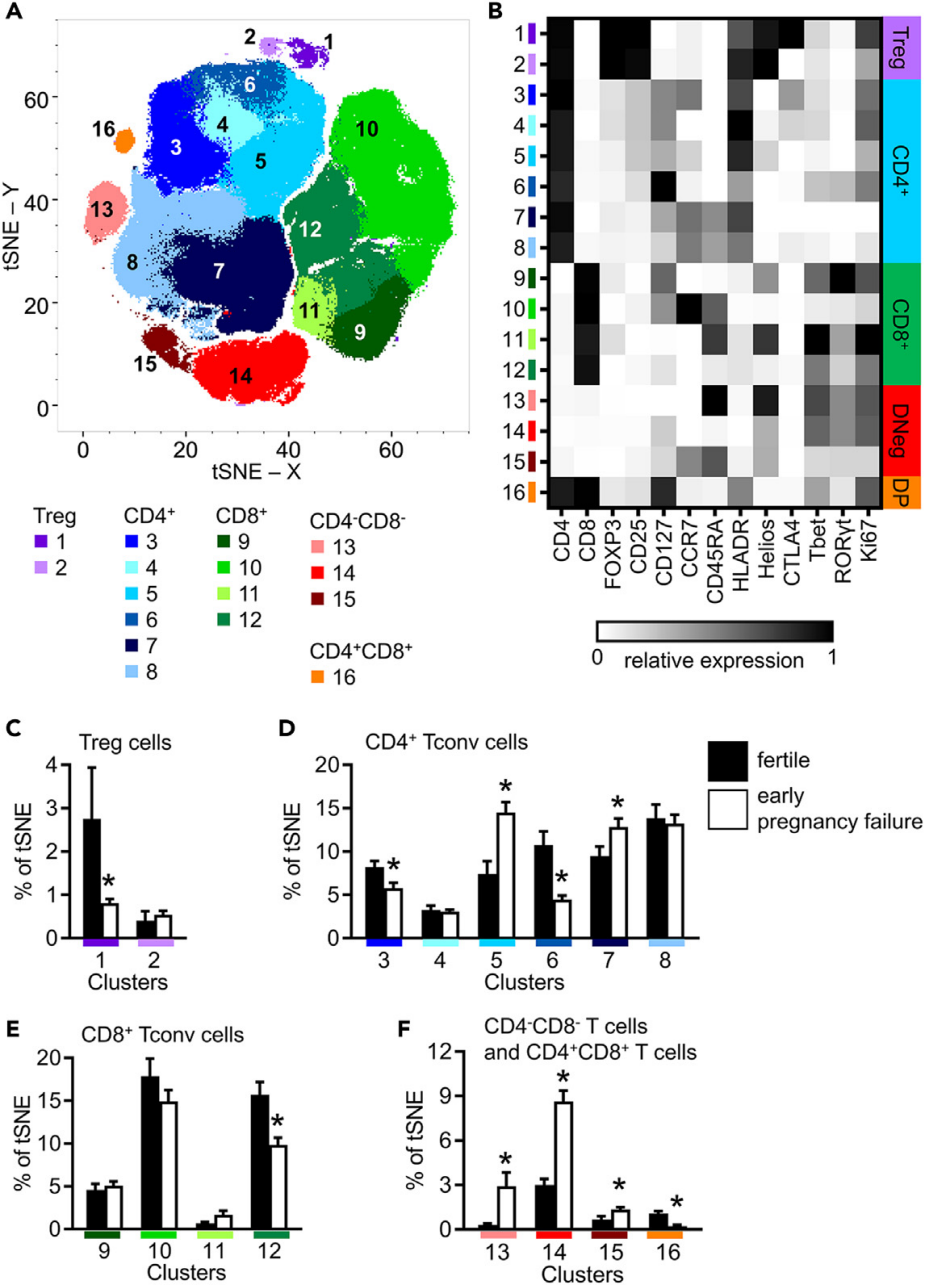
Unbiased t-distributed stochastic neighbor embedding (tSNE) and X-shift analysis of peripheral blood CD3^+^ T cells Peripheral blood T cells from proven fertile (Fert, n = 15) and early pregnancy failure subjects (EPF, n = 27) were analyzed by flow cytometry. A downsample of 7,344 CD3^+^ T cells from each sample were concatenated, visualized using the tSNE algorithm, and 16 unique cell clusters identified using X-shift analysis, which are represented by different colors (A). The relative mean fluorescence of each marker in each cluster was calculated and shown as a heatmap (B). Clusters were classified into subgroups of CD4^+^ Treg cells (C), CD4^+^ Tconv cells (D), CD8^+^ cells (E), and either double-negative CD4^−^CD8^−^ or double-positive CD4^+^CD8^+^ T cell (F) clusters, and the proportion of each cluster for each participant group calculated and shown as the mean ± SEM. The effect of fertility status was determined by t test. ∗p < 0.05.

t-distributed stochastic neighbor embedding (tSNE) utilizes an unsupervised, unbiased algorithm to visualize data using all marker information.^53^ Unbiased X-shift clustering^54^ using 7,344 CD3^+^ T cells from each participant identified 16 separate cell clusters among which CD4^+^ Treg cells, CD4^+^ Tconv cells, CD8^+^ Tconv cells, double-negative CD4^−^CD8^−^ T cells, and double-positive CD4^+^CD8^+^ T cell subsets were identified (Figure 4A). The MFI of 13 cell markers was determined for each of the 16 clusters (Figure 4B), and expression of each marker was mapped onto individual tSNE plots (Figure S4).

Two distinct Treg cell populations, Cluster 1 and Cluster 2, both showed high expression of CD4, FOXP3, CD25, and Helios, with low or negative expression of CD8 and CD127 (Figure 4B). Higher CTLA4 and Ki67 expression was evident in Cluster 1 than in Cluster 2 (Figures 4B and S4). Cluster 1 was substantially smaller in subjects with EPF compared to fertile subjects (Figure 4C), consistent with fewer CTLA4^+^ Treg cells seen in EPF using conventional gating (Figure 2), while the size of Cluster 2 was similar.

Six CD4^+^ Tconv clusters, Clusters 3–8 (Figures 4A and 4B), were identified by X-shift analysis. Cluster 3, consisting largely of T_EM_ cells (CCR7^−^CD45RA^−^) with moderate CD25, was reduced in EPF subjects (Figure 4D). Clusters 4 and 6 lacked expression of CD45RA and CCR7, suggesting these clusters comprise mainly T_EM_ cells (Figures 4A and 4B). Cluster 4 was unchanged between fertile and EPF subjects, while Cluster 6 was reduced in EPF subjects (Figure 4D). Cluster 5, a mixed population containing CCR7^−^CD45RA^−^ T_EM_ cells and CCR7^+^CD45RA^−^ T_CM_ cells (Figure 4A), was increased in EPF subjects (Figure 4D). For Clusters 7 and 8, both CCR7^+^CD45RA^+^ CD4^+^ T_naive_ cells with similar phenotypes, Cluster 7 was higher in EPF subjects compared to fertile subjects (Figure 4D).

CD8^+^ T cells (Figures 4A and 4B) were stratified into four clusters by X-shift analysis (Clusters 9–12, Figure 4). These clusters consisted primarily of CCR7^−^CD45RA^−^ T_EM_ cells (Clusters 9 and 12), CCR7^+^CD45RA^+^ T_naive_ cells (Clusters 10), and CCR7^−^CD45RA^+^ T_EMRA_ cells (Cluster 11, Figure 4B). Cluster 12 comprising CD8^+^ T_EM_ cells was reduced in EPF subjects, although Cluster 9 containing similar cells but with higher RORγt expression was comparable (Figure 4E).

X-shift analysis identified three clusters of double-negative CD4^−^CD8^−^ T cells (Figure 4A) (Clusters 13–15). Cluster 13 largely consists of CCR7^−^CD45RA^+^ T_EMRA_ cells, and Cluster 14 is a heterogeneous population of mainly CCR7^−^CD45RA^−^ T_EM_ cells, while Cluster 15 contains CCR7^+^CD45RA^+^ T_naive_ CD4^−^CD8^−^ T cells (Figures 4B and S4). Each of these was increased in EPF subjects, compared to fertile subjects (Figure 4F), consistent with the conventional 2D gating analysis (Figure 1). Finally, Cluster 16 (Figure 4A) containing double-positive CD4^+^CD8^+^ T cells (Figure 4B) was smaller in subjects with EPF (Figure 4F), mirroring the conventional analysis (Figure 1).

### tSNE analysis confirms impaired CD4^+^ Treg cells in EPF

**Figure 5:**
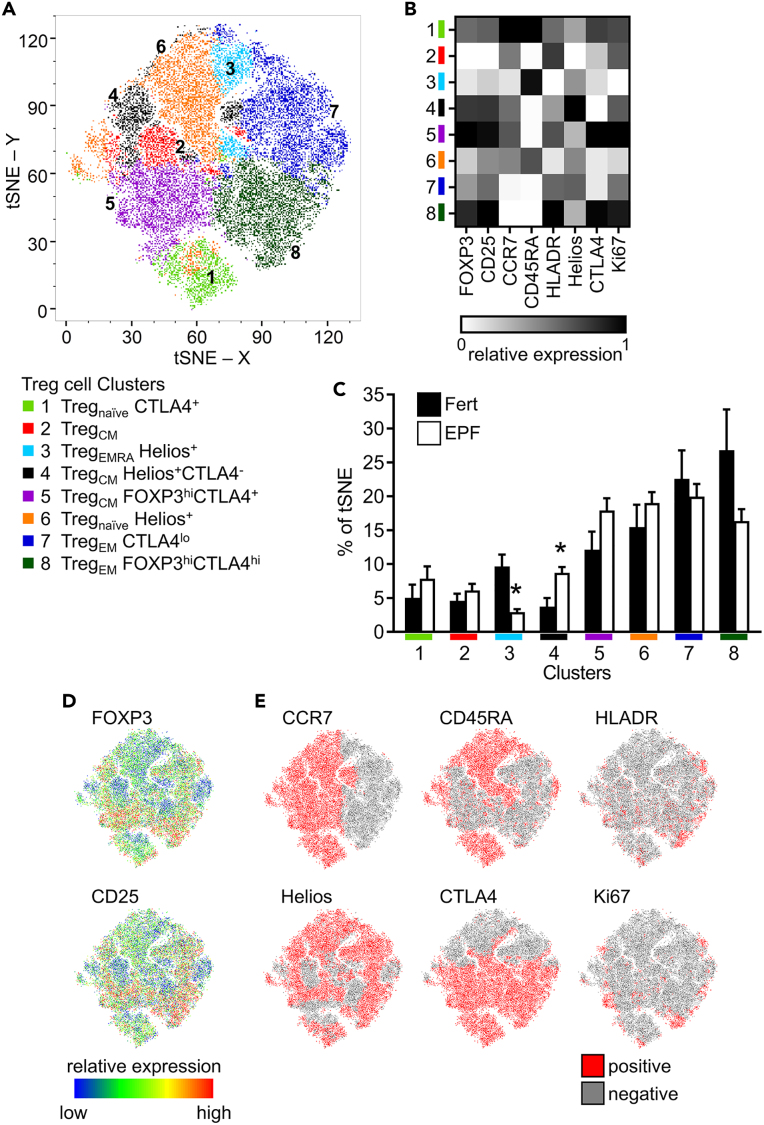
Unbiased t-distributed stochastic neighbor embedding (tSNE) and X-shift analysis of peripheral blood Treg cells Peripheral blood T cells from proven fertile (Fert, n = 15) and early pregnancy failure subjects (EPF, n = 27) were analyzed by flow cytometry. A downsample of 418 Treg (CD3^+^CD4^+^FOXP3^+^CD25^+^CD127^-/lo^) cells from each sample were concatenated, visualized using the tSNE algorithm, and 8 unique cell clusters identified using X-shift analysis, which are represented by different colors (A). The relative mean fluorescence of each marker in each cluster was calculated and shown as a heatmap (B). The proportion of each cluster for each Fert and EPF subject was calculated and shown as mean ± SEM (C), with the effect of fertility status determined by t test, ∗p < 0.05. The relative expression of FOXP3 and CD25 are shown on the tSNE plot as a heatmap (D) and Treg cells were manually gated for positive expression of CCR7, CD45RA, HLADR, Helios, CTLA4, and Ki67, with each marker then mapped onto the tSNE plot (E).

CD3^+^CD4^+^CD25^+^FOXP3^+^CD127^-/lo^ T cells from fertile and EPF subjects were also analyzed by tSNE and X-shift algorithms, to identify 8 separate Treg cell clusters (Figure 5A). The MFI of 8 Treg cell markers was determined for each cluster (Figure 5B). The relative expression of FOXP3 and CD25 (expressed by all Treg cells) is shown on tSNE plot heatmaps (Figure 5D), and Treg cells positive for CCR7, CD45RA, HLADR, Helios, CTLA4, and Ki67 are indicated on tSNE plots (Figure 5E).

The tSNE and X-Shift analysis of Treg cells confirmed aspects of the conventional 2D analysis. The proportions of 3 of the 8 clusters were altered in the EPF samples. Cluster 8, comprising Treg cells with strong expression of FOXP3, CD25, and CTLA4 and variable expression of HLADR and Ki67 (Figure 5B) indicating robust, highly suppressive, proliferating Treg cells, was reduced in the EPF group compared to the proven fertile controls (Figure 5C). Cluster 3 containing primarily CCR7^−^CD45RA^+^ Treg_EMRA_ cells with lower expression of FOXP3, CD25, HLADR, CTLA4, and Ki67 (Figure 5B) was markedly reduced in the EPF subjects (Figure 5C). This was counterbalanced by an increase in Cluster 4, containing mainly CCR7^+^CD45RA^−^ Treg_CM_ cells (Figure 5B), in EPF subjects (Figure 5C). The clusters that contained mostly CCR7^+^CD45RA^+^ Treg_naive_ cells, Clusters 1 and 6 (Figure 5B), were not different in EPF subjects (Figure 5C), but since Treg_naive_ cells did not cluster cleanly from other Treg cells, this analysis did not provide conclusive information on Treg_naive_ cells.

### RNA-seq in Treg and Tconv cells in EPF

**Figure 6:**
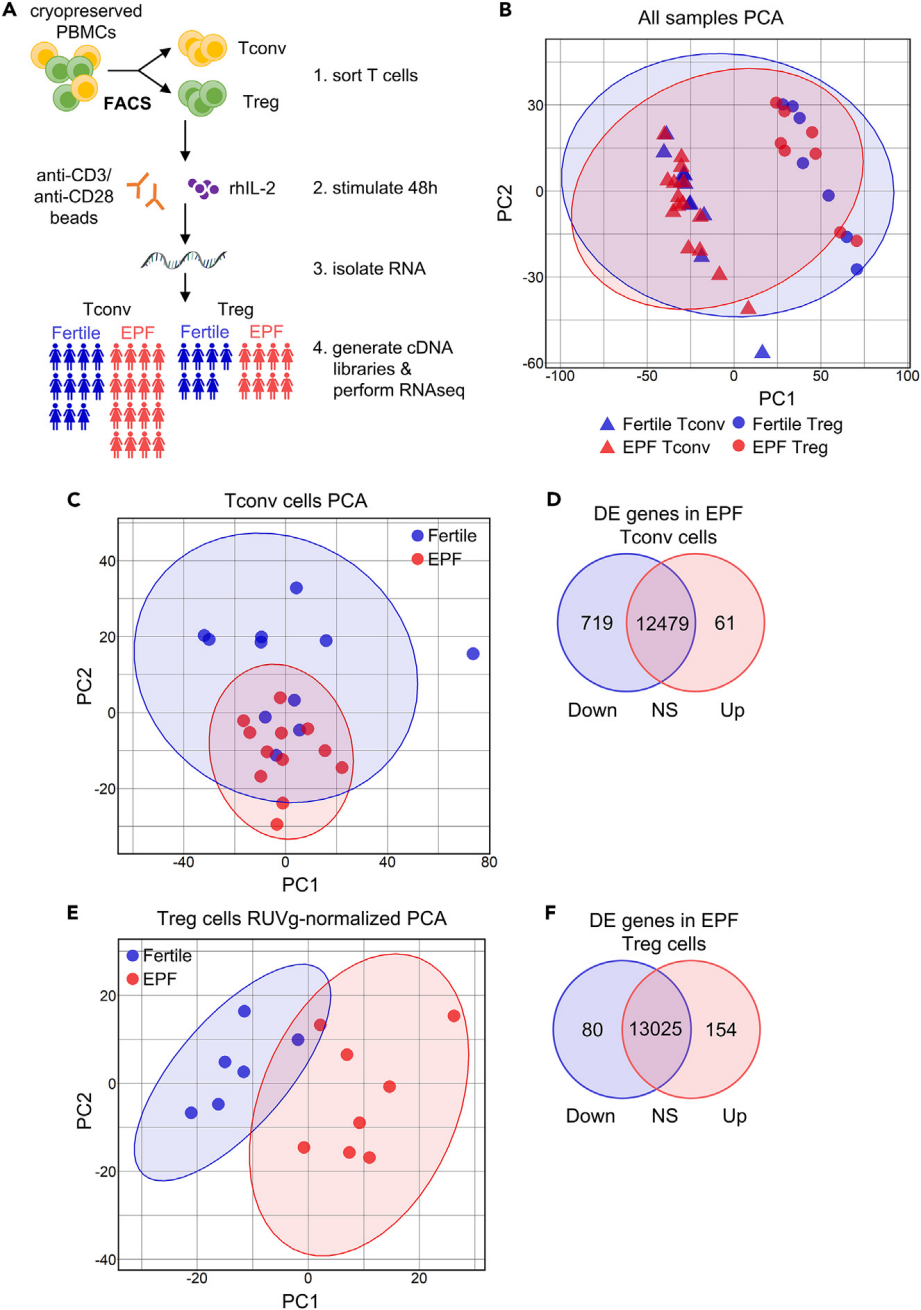
RNA-seq reveals altered transcriptome in Tconv cells and Treg cells in early pregnancy failure Tconv and Treg cells isolated from cryopreserved PBMCs from proven fertile and EPF subjects by FACS were stimulated *in vitro* for 48 h, before RNA was isolated and used to construct cDNA libraries for RNA-seq on an Illumina HiSeq platform (A). A principal-component analysis (PCA) plot for all Tconv and Treg cell RNA-seq samples, with ellipses showing the 95% CI for Tconv and Treg cell clustering around the mean (B). PCA plot shows Tconv cell samples from proven fertile (n = 11) and EPF subjects (n = 12) (C) and total differentially expressed genes identified in Tconv cells from EPF versus proven fertile subjects with an FDR-adjusted p < 0.05 are displayed in a Venn diagram (D). PCA plot shows Treg cell samples from proven fertile (n = 6) and EPF subjects (n = 8), after RUVg normalization (E). Total differentially expressed genes identified in Treg cells from EPF versus proven fertile subjects with FDR-adjusted p < 0.05 are displayed in a Venn diagram (F).

To evaluate whether T cell gene expression changes contribute to the altered T cell profile seen in EPF subjects, we isolated Treg and Tconv cells from cryopreserved PBMCs using FACS, stimulated the cells in culture, and then measured transcription by RNA-seq (Figure 6A). RNA-seq was performed for 27 Tconv cell samples (n = 11 for fertile and n = 16 for EPF) and 15 Treg cell samples (n = 7 for fertile and n = 8 for EPF). Due to low numbers of Treg cells causing a limited cell yield after FACS, there were fewer RNA-seq samples for Treg cells than Tconv cells. Using minimum expression criteria of ≥1 count per million (cpm) reads in at least 6 samples, the T cells were seen to express a total of 13,175 genes. The largest driver of variation in the data identified by principal-component analysis (PCA) was T cell type, causing Treg and Tconv samples to cluster separately on PC1 (Figure 6B).

Comparison of the differentially expressed (DE) genes between Treg and Tconv cells from EPF and fertile subjects revealed significant differences. In fertile subjects, 6,656 total DE genes, or 399 with a log-fold change (lfc) >1, equivalent to a 2-fold difference in expression, were identified between Treg cells and Tconv cells (Figure S5A). Among the most substantially upregulated DE genes were *FOXP3*, *IKZF2* (Helios), *TNFSF1B* (TNFR2), *CCR8*, *TOX*, *CTLA4*, *IL1R1*, and *IL1R2* and downregulated DE genes were *IL7R* and *IRF8* (Figure S5B), as expected.^29^ A similar number of 6,634 DE genes, or 396 with lfc >1, were identified for EPF Treg cells compared to EPF Tconv cells (Figure S5C). As in the fertile group, the DE genes included *FOXP3*, *IKZF2*, *CTLA4*, and *IL7R* (Figure S5D). However, only 64.4% of the genes DE in Treg versus Tconv cells overlapped between the fertile and EPF subjects (Figure S5E). To further investigate differences associated with fertility in the T cells, we proceeded to analyze the cell types separately.

### DE genes in Tconv cells in EPF

Three Tconv samples from the EPF group were excluded from the RNA-seq analysis when PCA showed they failed to cluster within the 95% confidence interval (CI) for the group (Figure S6A). After this, PCA showed overlapping clusters of fertile Tconv and EPF Tconv cells (Figure 6C). Differential gene expression (DGE) analysis was then performed with limma/voom,^55^ and, since cDNA libraries were prepared in batches, this was factored into the model to account for batch variation. 780 DE genes were identified in Tconv cells from the EPF group compared with proven fertile controls. Of these, most of the genes were downregulated (719 genes) whereas 61 genes were upregulated in EPF Tconv cells (Figures 6D and S6B).

Given our finding of increased pro-inflammatory Tconv cell responses in EPF subjects, the DE genes were screened for their association with inflammation or differentiation to specific Th subsets. The chemokine receptor *CCR4* is associated with Th2 cells and was increased in EPF Tconv cells. While *IL17RA*, a marker of inflammation, was increased in Tconv cells from EPF subjects, overall there was little evidence of increased pro-inflammatory function in the Tconv cell compartment in EPF subjects compared to fertile controls. In fact, some genes pointed to less effector activity, for example, decreased expression of the transcription factors *RUNX1*, *RUNX2*, and *RUNX3* (Table S3).

Minimal disruption to genes associated with T cell effector functions was mirrored in the DE gene pathway analysis for EPF Tconv cells. Over-representation analysis was performed to identify enrichment for gene ontology (GO) terms and Kyoto Encyclopedia of Genes and Genomes (KEGG) terms in the significantly DE gene sets for Tconv. The full list of Tconv DE genes yielded no significantly enriched GO or KEGG terms, so a log-fold change cutoff of 0.04 (equivalent to 1.03-fold difference in expression) was applied to narrow the results list to 241 DE genes for pathway analysis (Figure S6C). Tconv DE genes for EPF then showed enrichment for two GO terms, *histone methyltransferase complex* and *histone H3-K4 dimethylation* (Figure S6D).

To widen screening for patterns in the gene expression data, we employed gene set enrichment analysis (GSEA) using the Molecular Signatures Database (MSigDB) (Table S4). Some significantly enriched pathways were identified by GSEA, including upregulation of genes in the cell cycle pathways *Kong E2F3 targets* and *Zhou cell cycle genes in immune response at 6 h.* Additionally, pathways from the C7 Immune Signatures and C3 MicroRNA Targets (MIR) legacy databases were negatively enriched, indicating genes associated with those pathways were downregulated in EPF Tconv cells. Immune Signatures pathway analysis showed suppression of genes known to be upregulated in PBMCs after YF17D vaccination (GSE13485) or downregulated in PBMCs after influenza vaccination (GSE29617). Overall, there was little evidence for inflammatory dysfunction in the Tconv cells from EPF subjects.

### DE genes in Treg cells in EPF

**Figure 7:**
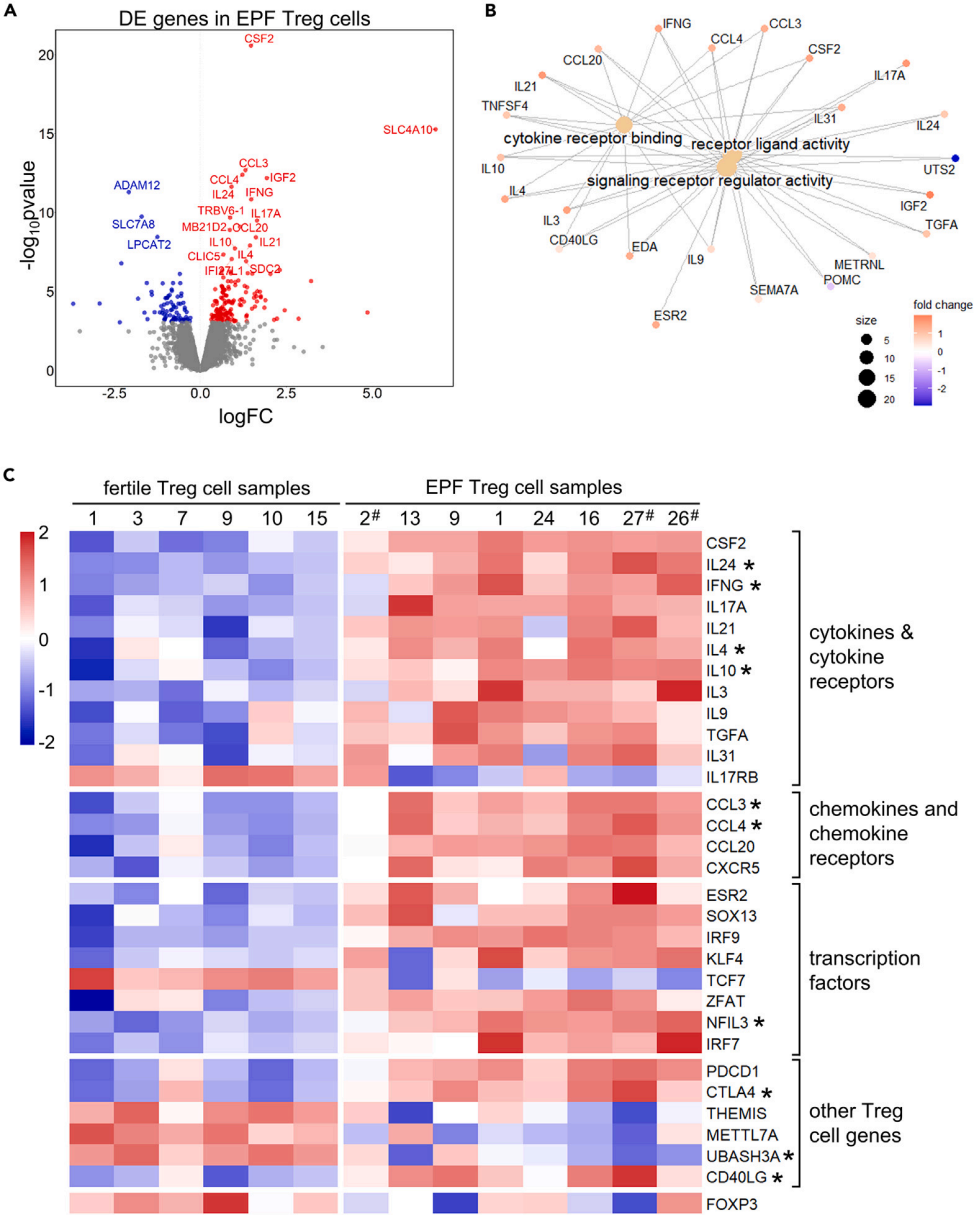
Differential expression of cytokines and immune-regulatory genes in Treg cells in early pregnancy failure A volcano plot displaying differentially expressed genes (DEGs) identified in Treg cells from proven fertile (n = 6) and EPF subjects (n = 8) was constructed (A). Significantly differentially expressed genes that are upregulated in EPF Treg cells are red, whereas downregulated genes are blue and the top 20 with the lowest adjusted p-values are labeled. Genes outside the *p* value cutoff for significance (Bonferroni-adjusted p < 0.05) are in gray. A GO network plot showing enriched Molecular Function terms and the associated differentially expressed genes for Treg cells from EPF compared with proven fertile subjects was constructed (B). The size of the dots for GO terms is larger when it is associated with more differentially expressed genes. The color of the dots indicates if the gene is upregulated (red) or downregulated (blue) in EPF Treg cells. Differentially expressed genes in Treg cells from proven fertile and EPF subjects that encode cytokines, cytokine receptors, chemokines, chemokine receptors, transcription factors, and other immune-regulatory molecules are displayed in a heatmap (C) showing relative expression in each sample based on *Z* scores calculated from the logCPM values. Red indicates the gene is upregulated relative to mean expression across all samples; blue indicates downregulation. ∗DEGs identified by in silico analysis to be targeted by FOXP3 transcription factor regulation are marked with asterisks. ^#^Individuals in the EPF group classified as positive for autoimmune status (see Table S2) are identified by hash symbol.

Initial PCA of Treg cell samples did not show clustering driven by fertility, and few significantly DE genes were identified in a first-pass limma/voom analysis (Figure S7A). Therefore we performed normalization using RUV (Remove Unwanted Variation) methodology to improve the detection of DE genes.^56^ For this, “*in silico* empirical” negative controls were formed using all but the top 5,000 genes ranked by edgeR p values, representing the least significantly DE genes. After RUV normalization together with exclusion of one proven fertile sample that failed to cluster with the rest of the group, Treg cell samples were seen to cluster by fertility status in the PCA plot (Figure 6E). Using the edgeR negative binomial generalized linear model (GLM) approach, 234 genes were identified as DE between Treg cells from the EPF and fertile groups, with 80 of these genes downregulated and 154 genes upregulated in Treg cells from the EPF group (Figures 6F and 7A; Table S5).

The expression level of selected genes that were significantly DE in Treg cells from EPF versus fertile subjects is shown for each sample in a heatmap (Figure 7C). Interestingly, 9 of the top 20 DE genes in Treg cells from the EPF group were cytokine genes with increased expression. Many of these cytokines are hallmark pro-inflammatory factors normally associated with highly activated and differentiated T helper cells, including *IFNG*, *IL4*, *IL17A*, and *CSF2*. This suggests Treg cells have compromised linage stability in EPF subjects. To investigate this, *FOXP3* expression was examined, and a trend to downregulated *FOXP3* expression in Treg cells from EPF compared to fertile subjects was found (Figure S8A, p = 0.108), consistent with the finding of reduced FOXP3 protein by flow cytometry (Figure 2C). The list of Treg cell DE genes was then screened for FOXP3 chromatin immunoprecipitation (ChIP) targets.^57^ Of the 234 DE genes in EPF Treg cells, 60 (25.6%) were FOXP3 ChIP hits, suggesting Treg cell gene dysregulation in EPF subjects is at least partly due to reduced FOXP3 (annotated in Table S5).

Other DE transcription factors in EPF Treg cells included upregulated estrogen receptor 2 (*ESR2*), SRY-box transcription factor 13 (*SOX13*), interferon regulatory factor 7 (*IRF7*), *IRF9*, and Kruppel-like factor 4 (*KLF4*) and downregulated transcription factor 7 (*TCF7*, encoding TCF1). Some of these changes would be expected to benefit Treg cell function, such as increased *ESR2,* as estrogen signaling enhances Treg cell expansion.^58^ Others, like *IRF7*, can have dual effects or negatively affect Treg cell function, like *IRF9* and *KLF4*. While transcription factor IRF7 binds to the *FOXP3* promoter and drives *FOXP3* expression,^59^ both IRF7 and IRF9 are positive regulators of type I interferon genes.^60^^,^^61^ KLF4, on the other hand, regulates *IL17* expression during Th17 differentiation.^62^

Dysregulation of both pro- and anti-Treg cell gene expression within Treg cells of EPF subjects is evident. Notably pro-inflammatory gene *TNF* was upregulated, as were anti-inflammatory genes including *IL10* and *CTLA4* and chemokines *CCL3* and *CCL4*, which are implicated in guiding CCR5^+^ effector CD4^+^ and CD8^+^ T cells toward Treg cells to enable their suppression.^63^ *CXCR5* and *PDCD1* (encoding PD-1), which are expressed by T follicular regulatory (Tfr) cells, were also upregulated. Although this might indicate increased Tfr differentiation, other genes normally associated with a Tfr signature such as *BCL6* and *ICOS* were not DE.

### Treg cell pathways implicated in EPF

Among the Treg cell DE genes, over-representation analysis revealed enrichment of 15 GO terms (Table S6) and 5 KEGG terms (Table S7). The most significantly over-represented GO term was related to the immune system, *hematopoietic or lymphoid organ development*. Other immune system-related GO terms for Treg cells included 3 related to cell movement (*regulation of locomotion*, *regulation of cell migration*, *myeloid leukocyte migration*) and 6 related to cell signaling (*signaling receptor regulator activity*, *receptor ligand activity*, *G protein-coupled receptor signaling pathway*, *positive regulation of tyrosine phosphorylation of STAT protein*), including 2 terms related to cytokine production and signaling (*cytokine production*, *cytokine receptor binding*) (Figure 7B). Terms in the cellular component ontology of GO terms were all related to the cell surface (*integral component of plasma membrane*, *plasma membrane region*, *cell surface*, *apical part of cell*).

GSEA was also performed for the Treg cell DE genes (for a full list of enriched terms, see Table S8). Increased T cell activation was inferred from the positive enrichment of *KEGG T cell receptor signaling pathway* and *primary immunodeficiency* (*PID*) *T cell receptor (TCR) calcium pathway* from MSigDB. The *PID IL23 pathway* was positively enriched, as was *hallmark TNFA signaling* via *NFKB*, providing further evidence for aberrant pro-inflammatory cytokine pathway activation in EPF Treg cells. Other enriched cytokine signaling pathways were *reactome interleukin-2 family signaling*, *marzec IL2 signaling up*, *reactome interleukin 10 signaling*, *KEGG cytokine-cytokine receptor interaction*, and *KEGG JAK STAT signaling pathway*. There was negative enrichment of many GSEA pathways for Treg cells from EPF subjects associated with cell cycle progression or proliferation, indicating downregulation of cell cycle-related genes. Enriched pathways associated with cell cycle included *Hallmark E2F targets*, *Hallmark G2M checkpoint*, *Ishida E2F targets*, *Kong E2F3 targets*, *Fischer G2 M cell cycle*, *Zhou cell cycle genes in immune response at 6 h (*and *24 h)*, and *Graham CML dividing* vs*. normal quiescent up* (Table S8).

We also investigated whether the genetic regions of the Treg cell DE genes were associated with particular diseases by performing enrichment analysis against the 2019 genome-wide association study (GWAS) database using the web-based tool Enrichr. Significant enrichment was identified for *Celiac disease*, *Type 1 diabetes*, and *Itch intensity from mosquito bite* (Table S9). Among the DE genes associated with these conditions were genes related to Treg cell regulatory function (*IL10*, *CTLA4*, *THEMIS*), negative regulation of TCR signaling (*UBASH3A*, *CTLA4*), and pro-inflammatory function (*IFNG*).

### Treg cell changes are independent of clinical evidence of autoimmunity

**Figure 8:**
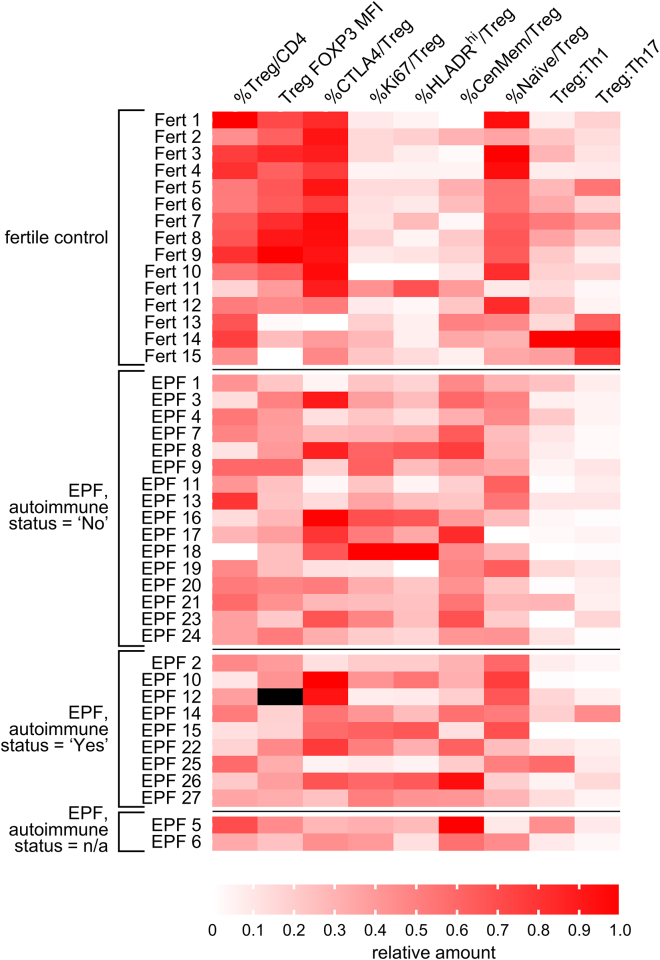
Impact of autoimmune status on Treg cell phenotype in early pregnancy failure The autoimmune status of 25/27 EPF subjects was designated “Yes” or “No” according to ESHRE clinical guidelines (see Table S2). Data on autoimmune status is not available (n/a) for proven fertile women or 2/27 EPF women. Values for flow cytometry parameters from proven fertile and EPF subjects are displayed in a heatmap showing relative score in each sample based on *Z* scores calculated from data shown in Figures 1, 2, and 3. Higher intensity of red indicates the parameter is higher relative to mean values across all samples. See Figure S9 for additional visualization of flow cytometry data according to autoimmune status.

The clinical guidelines of the European Society for Human Reproduction and Embryology (ESHRE) for the management of recurrent pregnancy loss^4^ recommend testing for autoantibodies (antinuclear, anticardiplin, B2-glycoprotein, and lupus anticoagulant) and thyroid function. Of the 27 EPF subjects, clinical data were accessible for 25, and 9 were positive for one or more autoimmune parameters, enabling classification of autoimmune status (Table S2). Analysis of flow cytometry data did not reveal a difference in Treg cell abundance, proliferation, or activation state according to autoimmune status, nor was there any change between the Treg:Th1 or Treg:Th17 ratios (Figures 8 and S9). Similarly, there was no evidence of association between autoimmune status and Treg cell defects as measured by transcriptional analysis (Figure 7C), including FOXP3 gene expression (Figure S8).

## Discussion

The degree to which a perturbed Treg cell pool contributes to EPF and the nature of the underlying defects are not well defined. Here we have used multi-colored flow cytometry and RNA-seq analysis to evaluate the prospect of a systemic shift in Treg cell phenotype. Collectively, the data reveal substantial changes in Treg cell abundance and phenotype, associated with transcriptional abnormalities indicative of impaired lineage stability and compromised immunosuppressive capacity, in a high proportion of EPF subjects. The Treg cell defects cause impaired constraint of pro-inflammatory cells indicated by increased RORγt^+^ Th17 cells, and substantially lower Treg:Th17 and Treg:Th1 cell ratios in EPF subjects. Since T cell populations residing in the uterine decidua largely originate from the peripheral blood and reflect systemic conditions,^11^^,^^23^ these changes have potential to disrupt pregnancy tolerance and impair embryo implantation. Importantly, altered Treg cell cellular and transcriptional parameters were evident in women experiencing EPF without identified autoantibodies or thyroid dysfunction, as well as those positive for indicators of autoimmunity.

The altered Treg cell profile in EPF was associated with skewing of the Treg cell population toward substantially fewer CCR7^+^CD45RA^+^ Treg_naive_ cells. Several biologically plausible scenarios may contribute. Naive Treg cells, like other T cells, are generated in the thymus before export via the blood to circulate through the body and secondary lymphoid organs.^64^ Encountering antigen triggers Treg cell differentiation to effector and memory cell subsets.^65^ Whereas Treg_CM_ continue to circulate, most Treg_EM_ and Treg_EMRA_ cells will egress to tissues to elicit their suppressive functions and die sooner than naive or central memory Treg cells.^66^ The causes of Treg_naive_ cell deficit therefore include (1) reduced *de novo* thymic generation; (2) reduced Treg_naive_ cell proliferation; (3) increased Treg_naive_ cell death; (4) increased Treg_naive_ cell egress and retention in tissues; (5) increased Treg_naive_ cell differentiation to effector or memory subsets; and/or (6) conversion Treg_naive_ cell to non-Treg cell subsets.

Our evidence strongly indicates lineage instability in Treg cells, which causes loss from the Treg cell pool during differentiation and conversion to effector T cell subsets.^48^ This interpretation is supported by the lower abundance of FOXP3 in Treg cells of EPF subjects, and their altered gene expression profile upon TCR stimulation. This is coupled with increased transcription of pro-inflammatory cytokines, including *IFNG*, *TNF*, *IL17A*, and *IL21*, and lower surface CTLA4 expression seen by flow cytometry. These findings concur with a previous study that reported reduced FOXP3 MFI in Treg cells, and less *FOXP3* mRNA, in women with unexplained recurrent pregnancy loss.^37^

The interpretation of reduced Treg cell stability in EPF is supported by reduced expression of Helios, a marker of Treg cell stability,^49^ in decidual Treg cells of miscarriage subjects.^27^ Demethylation of the Treg cell-specific demethylated region (TSDR) within the *FOXP3* promotor provides stable FOXP3 expression, leading to cells with strong suppressive capacity.^67^^,^^68^ In mice, there is further demethylation of this site in early pregnancy,^19^ implying Treg cell stability is important for pregnancy success. Increased methylation in the TSDR in unexplained miscarriage correlates with waning Treg cell abundance and suppressive capacity.^36^

Our data also point to a faster rate of Treg cell maturation contributing to decreased Treg cell abundance. Gene pathway analysis revealed transcriptional enrichment for TCR signaling pathways, suggesting that Treg cells from EPF women are hyper-responsive to TCR stimulation resulting in increased differentiation to effector and memory Treg cell subsets. A lower proportion of naive CD45RA^+^ Treg cells, with increased memory phenotype, is reported in women with unsuccessful *in vitro* fertilization (IVF) treatment.^69^ A similar change in Treg cell phenotype is observed in clinical conditions where immune dysregulation and breakdown of immune tolerance are implicated including multiple sclerosis^70^ and type 1 diabetes.^71^^,^^72^ The increase in *PDCD1* mRNA (encoding PD-1, CD279) in Treg cells from EPF subjects (Table S5) supports the interpretation of accelerated maturation and exhaustion in Treg cells. This aligns with previous findings of elevated exhaustion marker PD-1 on Treg cells from EPF^39^ and recurrent implantation failure^35^ subjects, and the possible benefit of intravenous immunoglobulin (IVIG), an experimental therapy for recurrent pregnancy loss^73^ that acts to reduce PD-1^+^ exhausted Treg cells in peripheral blood.^74^

If increased differentiation and conversion of Treg cells occur in EPF, this would increase demand on the supply of naive Treg cells. Thymic production of new Treg cells in humans declines after puberty so that in adults the peripheral pool of naive cells is maintained primarily by proliferation of mature naive (MN) Treg cells.^75^ Elevated Ki67 expression in EPF women might represent an attempt to maintain the pool of naive Treg cells. This is borne out in the observation that Treg cell proliferation was greatest in the EPF subjects exhibiting the lowest Treg cell abundancies (Figure S1A). Our results are reminiscent of the observation of increased proliferating Ki67^+^ Treg cells despite diminished Treg cells in the decidua from miscarried euploid embryos.^28^ There was no correlation between the number of pregnancy losses and the degree of Treg cell deficiency in EPF subjects (Figure S1G), similar to a previous report.^76^

In contrast to the flow cytometry data, RNA-seq GSEA analysis indicated suppression of cell cycle progression in EPF Treg cells (Table S8). This discrepancy likely reflects differential responses to *in vitro* stimulation of T cells prior to transcriptome analysis. *In vitro* activation is a standard approach to reveal the functional potential of T cells^77^^,^^78^ and is appropriate given early pregnancy exposes T cells to activating stimuli, but whether it authentically replicates *in vivo* conditions is unclear. Since proliferation was not increased in Treg_EM_ and Treg_CM_ cells (Figure S3) and cell cycle pathways were actually suppressed in EPF Treg cells, EPF Treg cells are unlikely to be inherently hyper-proliferative. Instead, the data support the interpretation that naive Treg cell proliferation is increased to maintain the naive Treg cell pool. It is possible that insufficient thymic generation of new Treg cells contributes to a depleted naive Treg cell pool in EPF, and future studies to quantify recent thymic emigrants (RTE) using T cell receptor excision circles (TREC) or CD31^79^ are warranted. The ratio of RTE:MN Treg cells determines the suppressive capacity of the total naive Treg cell pool^80^ and reasonably might be reduced in EPF.

Alternative explanations for reduced total Treg cells in the blood of EPF subjects are increased cell death or redistribution of the Treg cells to peripheral tissues. No evidence of increased cell death was identified in the transcriptional analysis. The lack of any change in chemokine receptor expression except for increased *CXCR5*, a marker of T follicular regulatory cells,^81^ does not support a change in egress from the blood. Nevertheless, regulation of cell migration and locomotion were enriched GO terms for EPF Treg cells, so further investigation of this possibility is warranted.

A key finding was reduced expression of CTLA4, implying that Treg cells from EPF women have impaired suppressive capacity. In fertile women, Treg cells were nearly uniformly 75%–90% CTLA4^+^, while a bimodal pattern of CTLA4 expression occurred in EPF women such that CTLA4^+^ expression correlated inversely with Treg cell proportion (Figure S1B). CTLA4 is a negative regulator of TCR signaling that can be induced by TCR hyper-stimulation in an attempt to constrain CD28 co-stimulation.^82^ Stimulation of Treg cells *in vitro* prior to cDNA library preparation upregulates *CTLA4*,^83^ potentially explaining the small upturn we saw in *CTLA4* expression in EPF women. We had insufficient cells to conduct suppression assays, the gold standard for measuring Treg cell functional competency, but this will be a priority in follow-up studies. Others have observed reduced Treg cell suppressive capacity in decidual Treg cells^26^ and peripheral blood Treg cells^22^ in EPF.

The EPF women in our study had an elevated proportion of HLADR^hi^ memory Treg cells, a population normally endowed with high suppressive capacity.^51^^,^^84^ EPF subjects with the fewest Treg cells tended to have the highest percentage of HLADR^hi^ Treg cells (Figure S1C). Collectively, increased Treg cell CTLA4, HLADR, and Ki67 expression suggests a compensatory attempt to counteract reduced Treg cell frequency. However this shift may be counterproductive, as women undergoing IVF showed higher suppressive activity of HLADR^+^ memory Treg cells when pregnancy failed.^69^ The possibility that Treg cells mature faster, leading to an earlier death, is supported by HLADR^hi^ Treg cells exhibiting greater proliferation (Figure S1D), and elevated susceptibility to apoptosis.^84^

A comparison of DE genes in the Treg cells from EPF subjects compared to fertile subjects demonstrated that EPF Treg cells had increased pro-inflammatory *IFNG*, *IL4*, *IL17A*, and *CSF2*. This may indicate their specialized differentiation to Treg cell subsets suited for suppression of cognate T helper subsets,^85^ or less phenotypic stability resulting in the adoption of pro-inflammatory signatures after stimulation, as would be expected with reduced FOXP3 protein. Treg cells from EPF subjects exhibited upregulated *KLF4*, a transcription factor which positively regulates *IL17*, using Th17 differentiation,^62^^,^^86^ and upregulation of *IRF7* and *IRF9*, key drivers of interferon gene and STAT1/STAT2 pathways.^60^^,^^61^^,^^87^

Reduced expression of *TCF7* (encoding the TCF1 protein) in EPF Treg cells is another indication of reduced phenotype stability. TCF1 interacts with FOXP3 to cooperate in regulation of genes important for Treg cell function.^88^^,^^89^ Treg cell-specific deletion of Tcf7 in mice shows it restrains excessive cell cycling and maintains suppressive function and may positively regulate Foxp3 expression.^89^ Downregulation of *TCF7* in Treg cells from EPF patients is thus likely to be detrimental. A regulatory link with *SOX13*, another transcription factor that was DE in EPF Treg cells, may exist. TCF1 acts as a transcriptional activator in the presence of β-catenin in canonical Wnt signaling,^88^ which is suppressed by SOX13. *SOX13* was upregulated in EPF Treg cells, which is likely to compound impaired *TCF7* expression to exacerbate Treg cell functional incompetence.

Other transcription factors expected to benefit Treg cell function were upregulated. One example is estrogen receptor *ESR2*, which mediates estrogen-mediated promotion of Treg cell induction, expansion, and stability.^58^ Other upregulated genes are anti-inflammatory in nature. *IL10* and the chemokines *CCL3* and *CCL4* have been implicated as a mechanism of guiding CCR5^+^ effector CD4^+^ and CD8^+^ T cells toward Treg cells to facilitate suppression.^63^ The upregulation of genes normally expected to improve Treg cell function indicates further compensatory adaptations.

There was little evidence of faults in the Tconv cell arm, and some gene changes indicate less effector activity. *RUNX1*, *RUNX2*, and *RUNX3*, genes involved in mature T cell function and differentiation to effector or memory subsets,^90^ were all downregulated in EPF Tconv cells. The increase in Th17 cells observed in EPF subjects and consequent decrease in the Treg:Th17 ratio presumably reflect insufficient Treg cell suppression and mirror earlier findings.^35^^,^^91^ Although *IL17RA* expression was increased in EPF Tconv and IL17 cells, IL17RA-mediated effects are linked to suppressive resistance of non-Th17 effector CD4^+^ T cells.^92^ Another observation of note was reduced CD4^+^CD127^hi^ cells in Tconv cells of EPF subjects. These cells have anti-inflammatory properties and are implicated in suppressing progression of type 1 diabetes.^93^

Many women with recurrent pregnancy loss and recurrent implantation failure have elevated anti-phospholipid, anticardiolipin, or other autoantibodies.^94^^,^^95^ We found that low Treg cell numbers, reduced FOXP3 MFI, and altered Treg cell transcriptional profiles were not linked with clinical indication of autoimmunity, and EPF women with autoantibodies did not exhibit worse Treg cell features. This suggests that Treg cell phenotype and transcriptional parameters may be more sensitive indicators of impaired pregnancy tolerance than autoantibody status.

Fewer naive Treg cells with impaired suppressive competence in peripheral blood is likely to be a constraint on availability of Treg cells to supply the endometrial compartment, given endometrial Treg cells are drawn from the blood most likely as naive cells that differentiate after extravasation.^23^^,^^24^ Activated effector Treg cells increase in both compartments during pregnancy^96^ and similar Treg cell deficits in blood and decidua in EPF.^97^^,^^98^^,^^99^^,^^100^^,^^101^ Nevertheless we acknowledge that specific Treg cell phenotypes are enriched in the endometrium, so that the endometrial Treg cell population is not identical to peripheral blood.^23^^,^^24^^,^^29^ Decidual Treg cells have a higher proportion of clonally expanded populations and enhanced suppressive capacity.^23^^,^^102^ A recent study reports differences in transcriptomes of Treg cells from blood and endometrium in EPF patients and indicates that endometrial Treg cells acquire a comparatively stronger regulatory profile and memory phenotype.^29^ An effort to identify differences in endometrial Treg cells in women with recurrent pregnancy loss revealed lower cell surface *TIGIT* and elevated *S1PR1* gene expression but found FOXP3, HELIOS, and CTLA4 were not different.^29^ The decrease in *TIGIT* expression is particularly interesting given the recent description of 3 distinct Treg subpopulations within the decidua: 1) CD25^hi^FOXP3^+^, 2) PD-1^hi^IL10^+^, and 3) TIGIT^+^FOXP3^dim^, all of which express high TIGIT^103^ and exhibit strong potential to suppress anti-fetal immunity.^103^^,^^104^ Future studies should therefore include analysis of both tissue compartments.

In summary, this study indicates that Treg cell dysfunction and compromised immune tolerance can arise in EPF due to an altered Treg cell transcriptional profile that impairs Treg cell suppressive competence. Altered FOXP3 expression leading to loss of Treg cell functional fitness and stability is implicated, and this may precede or be independent of synthesis of antibodies to common autoantigens. The similarity between the transcriptional changes seen in EPF and autoimmune conditions type 1 diabetes and celiac disease indicates the possibility of shared underlying Treg cell defects. These and other autoimmune conditions all appear to arise secondary to subtle alterations to the gene regulatory networks controlled by FOXP3.^47^^,^^85^^,^^105^ Our findings are consistent with emerging views that Treg cell instability is caused by single nucleotide polymorphisms (SNPs) within the FOXP3 gene body, or nearby noncoding regions or target genes, interacting with genetic disposition and precipitated by environmental and developmental factors.^47^ Collectively, our observations bolster the evidence that at least in some women EPF is due to underlying disruption in the Treg cell compartment and provide new insight on how this can arise at a genomic level. The findings strengthen the impetus for development of intervention strategies that modulate Treg cells for application in the reproductive medicine setting.^46^

### Limitations of the study

The current study is limited by the small study size and the heterogeneity of the EPF patients’ clinical histories, so it is not possible to extrapolate from the current findings to recurrent pregnancy loss and recurrent implantation failure more broadly. Although women meeting diagnostic criteria for recurrent pregnancy loss and recurrent implantation failure showed broadly similar Treg cell phenotypes to the EPF group as a whole (Figure S2), there were insufficient numbers for independent transcriptome analysis. The considerable between-individual variance in transcriptomic profile indicates that not all women with EPF have abnormal Treg cells, and, within those that do, a range of degrees and types of Treg cell dysfunction likely exist. A larger study is now required to determine what proportion of EPF is associated with altered Treg cell features, to investigate the heterogeneity in Treg cell phenotypes and relationships with age and clinical features, and to evaluate transcriptional differences that may distinguish different clinical categories.

Another limitation is the lack of information on genetic status of lost embryos. Around 50% of miscarriages involve embryo aneuploidy, when loss is considered a normal physiological response. Aberrant decidual Treg cells are more apparent in women with loss of normal euploid embryos, compared to aneuploid embryos.^27^^,^^28^ In this study, at least a proportion of the losses in some subjects reasonably would have been aneuploid. Nevertheless, given that 19 of the 27 patients had 3 or more losses, and of the 8 patients with 2 losses only one was aged over 40 years, we expect this to be a low proportion. Future research, in a larger population wherein embryo genetic information is available, is required to confirm these findings.

## STAR★Methods

### Key resources table

**Table:** 

REAGENT or RESOURCE	SOURCE	IDENTIFIER
**Antibodies**		

BUV395 anti-human CCR7 (CD197) antibody	BD Biosciences	Cat# 740267; RRID: AB_2740009
APC-H7 anti-human CD3 antibody	BD Biosciences	Cat# 560176; RRID:AB_1645475
BUV496 anti-human CD4 antibody	BD Biosciences	Cat# 564651; RRID: AB_2744422
BUV737 anti-human CD8 antibody	BD Biosciences	Cat# 612754; RRID:AB_2870085
BV786 anti-human CD25 antibody	BD Biosciences	Cat# 741035; RRID: AB_2740652
PE-Cy™7 anti-human CD127 antibody	BD Biosciences	Cat# 560822; RRID: AB_2033938
BB515 anti-human CD45RA antibody	BD Biosciences	Cat# 564552; RRID:AB_2738841
BV510 anti-human HLA-DR antibody	BD Biosciences	Cat# 563083; RRID: AB_2737994
PE-CF594 anti-human FoxP3 antibody	BD Biosciences	Cat# 563955; RRID:AB_2738507
Alexa Fluor® 647 anti-Helios antibody	BD Biosciences	Cat# 563951; RRID:AB_2738506
PE-Cy™5 anti-human CD152 (CTLA4) antibody	BD Biosciences	Cat# 561717; RRID: AB_10893816
Alexa Fluor™ 700 Ki67 antibody, eBioscience	Thermo Fisher Scientific	Cat# 56-5698-82; RRID:AB_2637480
PE anti-human RORγt antibody	BD Biosciences	Cat# 563081; RRID:AB_2686896
BV421 anti-Tbet antibody	BD Biosciences	Cat# 563318; RRID: AB_2687543

**Chemicals, peptides, and recombinant proteins**		

Recombinant human IL2	Peprotech, Rehovot, Israel	#200-02-10
DNase I	Worthington, Biochemilca, Lakewood, NJ	#LS002007
Gibco™ GlutaMAX™ supplement	Thermo Fisher	#35050061
HEPES sodium salt	Sigma Aldrich	#H3784-100G
Sigma Antibiotic Antimycotic solution (100x)	Sigma Aldrich	#A5955-100ML

**Critical commercial assays**		

RNeasy Plus Micro Kit	Qiagen, Hilden, Germany	#74034
eBioscience™ Foxp3/Transcription Factor Staining Buffer Set	Thermo Fisher	#00-5523-00
NEBNext® Poly(A) mRNA Magnetic Isolation Module	New England Biolabs, Ipswich, MA	#E7490L
NEBNext® Ultra™ II Directional RNA Library Prep Kit for Illumina®	New England Biolabs, Ipswich, MA	#E7760L
NEBNext® Multiplex Oligos for Illumina®	New England Biolabs, Ipswich, MA	#E6609S
Universal KAPA Library Quantification Kit	Roche, Basel, Switzerland	#KK4824

**Deposited data**		

RNAseq data	NCBI GEO	GSE239865

**Software and algorithms**		

GraphPad Prism v9.0.0	GraphPad Software Inc.	www.graphpad.com
FlowJo v10.6.2	BD Biosciences/FlowJo, LLC	www.flowjo.com
tSNE FlowJo plugin	BD Biosciences/FlowJo, LLC	www.flowjo.com/exchange
XShift FlowJo plugin	BD Biosciences/FlowJo, LLC	www.flowjo.com/exchange
R v4.2.1	The R foundation	www.r-project.org
RStudio v2022.02	RStudio	www.rstudio.com
Molecular Signatures Database (MSigDB) v7.0 (human)	UC San Diego and Broad Institute	www.gsea-msigdb.org/gsea/msigdb
Enrichr and built-in 2019 GWAS database	Maayan lab	maayanlab.cloud/Enrichr/

**Other**		

Human Lympholyte Cell Separation Media	Cedarlane, Burlington, Canada	#CL5020
Fixable Viability Stain 575V	BD Biosciences	#565694
Brilliant Staining Buffer Plus	BD Biosciences	#566385
Human BD Fc Block	BD Biosciences	#564220
UltraComp eBeads™ Compensation Beads	Thermo Fisher	#01-2222-42
BD CompBeads Set Anti-Mouse Ig,κ	BD Biosciences	Cat# 552843; RRID: AB_10051478
Gibco™ Dynabeads Human T-Expander CD3/CD28	Thermo Fisher	#11141D
SPRIselect PCR clean-up beads	Beckman Coulter	#B23318
Gibco™ RPMI-1640 Medium, no phenol red	Thermo Fisher Scientific	#11835030
X-VIVO medium	Lonza Bioscience, Basel, Switzerland	#04-418Q
Dulbecco’s modified phosphate-bufferred saline	Sigma-Aldrich	#D8537

### Resource availability

#### Lead contact

Further information and requests for resources and reagents should be directed to and will be fulfilled by the lead contact, Sarah A. Robertson (sarah.robertson@adelaide.edu.au).

#### Materials availability

This study did not generate new unique reagents.

#### Data and code availability

• RNAseq data have been deposited at the National Center for Biotechnology Information’s Gene Expression Omnibus. The accession number is listed in the key resources table and data are publicly available as of the date of publication. Flow cytometry data and deidentified clinical data reported in this paper will be shared by the lead contact upon request.
• This paper does not report original code.
• Any additional information required to reanalyze the data reported in this paper is available from the lead contact upon request.

### Experimental model and study participant details

#### Ethics approval and study design

Approval for the study was obtained from the Human Research Ethics Committees at the University of Adelaide (approval numbers H-2020-014 and H-2013-037), and St. Andrews Hospital Adelaide (REC2439/12/14). Subjects were women (adults assigned female sex at birth) who had experienced early pregnancy failure (n = 27), and women with proven fertility (n = 15). All participating subjects were Australians of European or Asian ancestry, aged 26 to 45 years, who provided informed written consent and had no serious underlying health conditions. Early Pregnancy Failure (EPF) subjects met the inclusion criteria of 2 or more miscarriages and/or embryo implantation failures following embryo transfer in an IVF treatment cycle. EPF patients had no diagnosed cause for pregnancy loss. EPF subjects were further designated as recurrent pregnancy loss (n = 11), when subjects had two or more miscarriages,^2^^,^^4^ and/or recurrent implantation failure (n = 10), when clinical pregnancy was not achieved after the transfer of four or more good quality embryos (day 5, early blastocyst stage), in three or more IVF treatment cycles.^3^^,^^6^ Two patients fit the definition of both recurrent pregnancy loss and recurrent implantation failure. Proven fertile subjects had all given birth to one or more live infants. EPF subjects had all experienced their most recent pregnancy loss at least 6 weeks and not more than 12 months prior to blood sample collection. Information on the genetic status of lost embryos was unavailable. Autoantibody parameters (titers of antinuclear antibody, ANA; anticardiolipin, ACA; B2-glycoprotein, B2GP; lupus anticoagulant, LA, and thyroid-stimulating hormone) were extracted from clinical records and were available for 25/27 EPF subjects (25/27) but were not available for proven fertile (Fert) subjects. ANA+ = 1:80; ANA++ = 1:160; ANA+++ = 1:640; LA+ = positive, TSH+ = > 4.5 mIU/L, HT = Hashimoto’s thyroiditis. Autoimmune status was designated ‘Yes’ or ‘No’ according to ESHRE guidelines recommending testing α-nuclear Ab, α-phospholipid Abs, and thyroid function. Exclusion criteria were use of any immune-modulating drugs (corticosteroids, NSAIDs, or methotrexate), evidence of an infection pathology, or use of hormonal contraception. Study participant clinical details are provided in Tables S1 and S2.

### Method details

#### Peripheral blood mononuclear cell collection and isolation

Peripheral venous blood was collected by venepuncture on the median cubital vein between 1000 h and 1400 h at the mid-luteal phase of the menstrual cycle in EDTA-coated Vacutainer tubes (BD Biosciences, Franklin Lakes, NJ). All women were naturally cycling and were not engaged in reproductive medicine treatment cycles or taking hormones or other fertility medication in the cycle when blood was collected. Within 4 h of sample collection, blood was centrifuged at 2000 x g for 10 min at room temperature (RT), and plasma was removed. Dulbecco’s modified phosphate-buffered saline (DPBS, Sigma-Aldrich, St. Louis, MO) was then added in a volume equal to the plasma removed, then further DPBS was added to the sample to double the volume, before layering over Lympholyte-H cell separation media (Cedarlane Laboratories, Burlington, Canada) and centrifugation at 600 x g for 30 min at RT without brake. The PBMC-containing layer was collected, washed twice in DPBS, centrifuged at 450 x g for 5 min and cells were resuspended in complete RPMI-1640 media (RPMI, Thermo Fisher) containing 10% FBS (Stemcell Technologies, Vancouver, Canada) and antibiotic and antimycotic (Sigma Aldrich). Cells were labeled with trypan blue (Sigma-Aldrich, St Louis, MO) and total viable cells were counted using a hemocytometer. Cell concentration was adjusted to ∼6 × 10^6^ cells per mL in complete media, with an equal volume of freezing media (20% DMSO [Sigma-Aldrich] and 80% FBS) added dropwise. Aliquots containing ∼3 × 10^6^ PBMCs, were stored in cryovials and frozen at a controlled rate to −80°C in a Mr Frosty (Nalgene, Rochester, NY) overnight. The following day PBMC cryovials were transferred into liquid nitrogen storage.

#### Flow cytometry

Cryopreserved PBMC samples were thawed, washed in complete RPMI and counted using a haemocytometer and trypan blue. Cells were rested overnight under cell culture conditions in a 96 well plate at 1 × 10^6^/mL. The following day, cells were washed in PBS and incubated with fixable viability stain 575V (1/1000 dilution) and human Fc block (1/30 dilution) in PBS for 10 min. Without washing cells, CCR7—BUV395 antibody was added and allowed to incubate for 15 min, before the addition of the surface-staining antibody master mix (CD3—APC-H7, CD4—BUV496, CD8—BUV737, CD25—BV786, CD127—PE-Cy7, CD45RA—BB515 and HLADR—V510, more details in Table S10), prepared in Brilliant Staining Buffer Plus (BD Biosciences) and a further 20 min incubation. Cells were washed in PBS and then fixed and permeabilized using the FOXP3 Transcription Factor Staining Buffer Set (Thermo Fisher) according to the manufacturer’s instructions. Cells were washed in permeabilization buffer and then incubated for 40 min at RT with a master mix of intracellular-staining antibodies (FOXP3—PECF594, Helios—AF647, CTLA4—PE-Cy5, Ki67—AF700, RORγt—PE, Tbet—BV421, more details in Table S10) prepared in Brilliant Staining Buffer Plus. All incubations were performed in the dark. Cells were washed and resuspended in permeabilization buffer, and analyzed on a BD LSR Fortessa X20 flow cytometer with FACSDiva software (BD Biosciences). In each experiment fluorescence minus one (FMO) and unlabelled controls were run to assist with gating positive and negative populations and single color compensation controls were performed utilizing either BD anti-mouse Igκ CompBeads (BD Biosciences) or UltraComp eBeads (Thermo Fisher).

#### Flow cytometry data analysis

All flow cytometry data was analyzed using FlowJo software (BD Biosciences, version 10.6.2). Gates were established to exclude dead cells, debris and doublets, and assess subpopulations of viable CD3^+^ T cells (gating strategy in Figure S10). 2-dimensional flow cytometry plots were used to identify CD4^+^, CD8^+^, CD4^+^CD8^+^ and CD4^-^CD8^-^ T cells. Treg cells were defined as CD3^+^CD4^+^CD25^+^FOXP3^+^CD127^−/lo^ and were further assessed for phenotype, using Helios, CTLA4, HLADR, and CD45RA, surrogate markers for Treg cell functional capacity, and using Ki67 as a marker of proliferation. Non-Treg CD4 Tconv and CD8 Tconv populations were analyzed for Tbet and RORγt expression to identify Th1 and Th17 T cells, respectively. All T cell compartments were assessed for the expression of CCR7 and CD45RA to identify naive (T_na___i___ve_, CCR7^+^CD45RA^+^), central memory (T_CM_, CCR7^+^CD45RA^−^), effector memory (T_EM_, CCR7^−^CD45RA^−^) and terminally differentiated effector memory expressing CD45RA (T_EMRA,_ CCR7^−^CD45RA^+^)^52^ phenotypes. Data are presented as the percentage of cells expressing specific markers or the geometric mean fluorescence intensity (MFI) of an individual marker within a specified population.

Flow cytometry data were also analyzed using the nonbiased, nonlinear dimensionality reducing t-distributed stochastic neighbor embedding (tSNE) algorithm^53^ and clustering algorithm, X-shift.^54^ Firstly, viable, singleton CD3^+^ T cells from the 42 samples (15 fertile and 27 EPF) were gated (Figure S10). Events with extreme fluorescence (negative or positive) were excluded. A downsample of 7,344 viable CD3^+^ T cells from each sample was concatenated into a single file. The tSNE was constructed in FlowJo taking into account the fluorescence of CD4, CD8, FOXP3, CD25, CD127, CCR7, CD45RA, HLADR, Helios, CTLA4, Tbet, RORγt and Ki67, using 1000 iterations of the Barnes-Hut algorithm, with a perplexity of 30 and learning rate of 1000. X-shift clustering analysis^54^ was performed using expression of the same 13 markers, with the *k* nearest neighbor default value of 102, distance metric set at Euclidean and a 100,000 cell subsampling limit set.

A second tSNE was constructed using viable CD3^+^CD4^+^FOXP3^+^CD25^+^CD127^-/lo^ Treg cells. Each sample had 418 Treg cells concatenated into a single file and a tSNE plot generated using the fluorescence of CCR7, CD45RA, HLADR, Helios, CTLA4 and Ki67, using 1000 iterations of the Barnes-Hut algorithm, with a perplexity of 30 and learning rate of 870. X-shift clustering analysis was performed using expression of the same 6 markers, with the *k* nearest neighbor value of 222 and distance metric set at Euclidean.

For each cluster in each of the two tSNE plots the MFI of each marker was determined and relative expression calculated. The proportion of cells within each cluster of the tSNE plot were assessed for each sample and the mean and standard deviation calculated. For both the CD3^+^ and Treg cell tSNE, each marker of interest was manually gated and applied to the tSNE plot to visualize positively labeled cells.

#### T cell isolation and stimulation for RNAseq

Cryopreserved PBMCs were thawed and resuspended in PBS for staining. The cells were blocked with Fc block and stained with viability dye (FVS 575V) for 10 min before adding antibodies against CD3, CD4, CD25 and CD127, and incubating for a further 20 min at RT. They were rinsed and resuspended in RPMI +10% FCS for sorting. CD4^+^ Treg (CD3^+^CD4^+^CD25^+^CD127^-/lo^) and CD4^+^ Tconv (CD3^+^CD4^+^CD25^+/−^CD127^+^) cells were gated (Figure S10F) and isolated by fluorescence-activated cell sorting (FACS) using a BD FACSAria Fusion cell sorter into complete X-VIVO medium [X-VIVO medium (Lonza Bioscience, Basel, Switzerland) supplemented with 10% FCS, 1% Glutamax (Thermo Fisher), 1 mM HEPES (Sigma-Aldrich) and 1% Antibiotic-Antimicotic (Thermo Fisher)]. After sorting, cells were resuspended in complete X-VIVO media and rested for 2 h at 37°C before stimulation with Dynabeads Human T-Expander CD3/CD28 (Thermo Fisher) at a 1:1 ratio in complete X-VIVO containing 400 U/mL rhIL2 (Peprotech, Rehovot, Israel).^77^^,^^78^ After 48 h at 37°C, 5% CO_2_, cells were harvested by incubating with 200 U/mL DNase I (Worthington Biochemilca, Lakewood, NJ) for 30 min at 37°C before removing the Dynabeads by magnetic separation and rinsing with PBS. The cells were counted, homogenized in Buffer RLT Plus (Qiagen RNeasy Micro Kit, Hilden, Germany) and stored at −80°C.

#### RNA isolation and RNAseq library preparation

RNA was isolated from the frozen T cell lysates using the RNeasy Micro Plus Kit (Qiagen). The RNA was quantified using a NanoDrop One spectrophotometer (Thermo Fisher) and the quality was checked with an Agilent Bioanalyzer (Santa Clara, CA) at the Australian Genome Research Facility (AGRF, Adelaide, Australia) or an Experion (Bio-Rad, Hercules, CA). cDNA libraries were generated using the NEBNext Poly(A) mRNA Magnetic Isolation Module (New England Biolabs, Ipswich, MA), NEBNext Ultra II Directional RNA Library Prep Kit from Illumina (San Diego, CA) and NEBNext Multiplex Oligos for Illumina, according to the manufacturer’s protocols. The libraries were quantified with the Universal KAPA Library Quantification Kit (Roche, Basel, Switzerland) and those with sufficient DNA (≥2 nM) were pooled to equimolar concentration. Paired-end 150bp sequencing was performed at GENEWIZ (Azenta Life Sciences, Chelmsford, MA) on an Illumina Hi-Seq platform. Libraries were sequenced to an average read depth of 56.3 million reads per sample.

#### RNAseq data analysis

Read quality was checked with FastQC v0.11.7 (Babraham Bioinformatics, Cambridge, UK) before and after adapter trimming with *AdapterRemoval* v2.2.1. Reads were aligned to the GRCh38 genome using *STAR* v2.5.3 and counted with *Subread* v1.5.2. Differential gene expression analysis was performed in R v4.2.1 and R studio v2022.02 with *limma* v3.52.2, *RUVseq* v1.30.0 and *edgeR* v3.38.1 packages. Prior to analysis of differentially expressed genes, 3 Tconv samples lying outside the 95% CI for the EPF group, and 1 Treg sample lying outside the 95% CI for the proven fertile group, were excluded. Correction for multiple comparisons utilised Bonferroni adjustment or the 2-stage step-up method of Benjamimi, Krieger and Yekutieli^106^ as specified. A false discovery rate (FDR) cut-off of 0.05 was used to determine statistical significance with no specified log fold-change (logFC) threshold, or logFC >0.04 for Tconv cells, prior to pathway analysis. A heatmap of gene expression was generated using the package *pheatmap* v1.0.12 in R. RNAseq data are deposited at the National Center for Biotechnology Information’s GEO: GSE239865.

#### Pathway analysis, and GWAS enrichment analysis

Pathway analysis for gene ontology (GO) and KEGG term enrichment was performed using hypergeometric testing with the R package *clusterProfiler* v4.4.4 and redundant terms were removed with the *simplify* function of *clusterProfiler*. Gene set enrichment analysis (GSEA) was performed with *fgsea* v1.22.0 using the Molecular Signatures Database (MSigDB) v7.0.^107^^,^^108^ Analysis for enrichment of differentially expressed genes common to autoimmune conditions was performed with the EnrichR online tool using the built-in 2019 GWAS database (maayanlab.cloud/Enrichr/). In all analyses, significance was assigned when Bonferroni-adjusted p < 0.05. Analysis utilized supercomputing resources provided by the Phoenix HPC service at the University of Adelaide.

### Quantification and statistical analysis

All graphs and flow cytometry heat maps were constructed in GraphPad Prism (GraphPad Software, San Diego, California USA, version 9.0.0). Statistical analysis of flow cytometry data was conducted in GraphPad Prism. Datasets were tested for normality of distribution by Shapiro–Wilk and Kolmogorov-Smirnov tests, then analyzed using unpaired t-test, or ANOVA and post-hoc t-test for comparison of multiple groups, to evaluate the effect of fertility status. The effect of multiple comparisons was corrected using FDR correction,^106^ and an effect of fertility status was considered significant when FDR-adjusted p < 0.05. A small number of data points (0–1 per parameter) defined as definitive outliers by both ROUT test (Q = 0.1%) and Grubb’s test (p < 0.01) were excluded from the analysis. Statistical significance is indicated in Figures with asterisks: ∗p < 0.05, ∗∗p < 0.01, ∗∗∗p < 0.001. Statistical analysis of RNAseq data is detailed above.

### Additional resources

No additional resources were utilized.

## Supplemental information

Document S1. Figures S1‒S10 and Tables S1, S2, S4, and S6‒S10

Table S3. Differentially expressed genes in Tconv cells from early pregnancy failure subjects compared with Tconv cells from fertile subjects, related to Figure 6

Table S5. Differentially expressed genes in Treg cells of early pregnancy failure subjects compared to Treg cells with fertile subjects, related to Figure 6
