# Supplementary material for: A disrupted FOXP3 transcriptional signature underpins systemic regulatory T cell insufficiency in early pregnancy failure
Source: iScience. 2024 Jan 23;27(2):108994. doi: 10.1016/j.isci.2024.108994 (PMC10847744; doi:10.1016/j.isci.2024.108994)
Supplement: Document S1. Figures S1‒S10 and Tables S1, S2, S4, and S6‒S10 [file mmc1.pdf]

iScience, Volume *27*

Supplemental information

A disrupted FOXP3 transcriptional signature underpins systemic regulatory T cell insufficiency in early pregnancy failure

Lachlan M. Moldenhauer, Kerrie L. Foyle, Jasmine J. Wilson, Ying Y. Wong, David J. Sharkey, Ella S. Green, Simon C. Barry, M. Louise Hull, and Sarah A. Robertson

Supplementary Information for

A disrupted FOXP3 transcriptional signature underpins systemic regulatory T cell insufficiency in early pregnancy failure

Authors: Lachlan M. Moldenhauer[1]*, Kerrie L. Foyle[1]*, Jasmine J. Wilson[1], Ying Y. Wong[1], David J. Sharkey[1], Ella S. Green[1], Simon C. Barry[1], M. Louise Hull[2], and Sarah A. Robertson[1‡]

[1]Robinson Research Institute and Adelaide Medical School, The University of Adelaide, Adelaide, SA 5005, Australia.

[2]School of Biological Sciences, University of Adelaide, Adelaide, SA 5005, Australia.

Corresponding author:

Sarah A Robertson, PhD, Robinson Research Institute, Adelaide School of Medicine, University of Adelaide, Adelaide Health and Medical Sciences Building, North Terrace, Adelaide SA 5005, Australia, T: +61 8 8313 4094, E-mail: sarah.robertson@adelaide.edu.au

This PDF file includes:

Supplementary Figures S1 to S10

Supplementary Tables S1 to S10

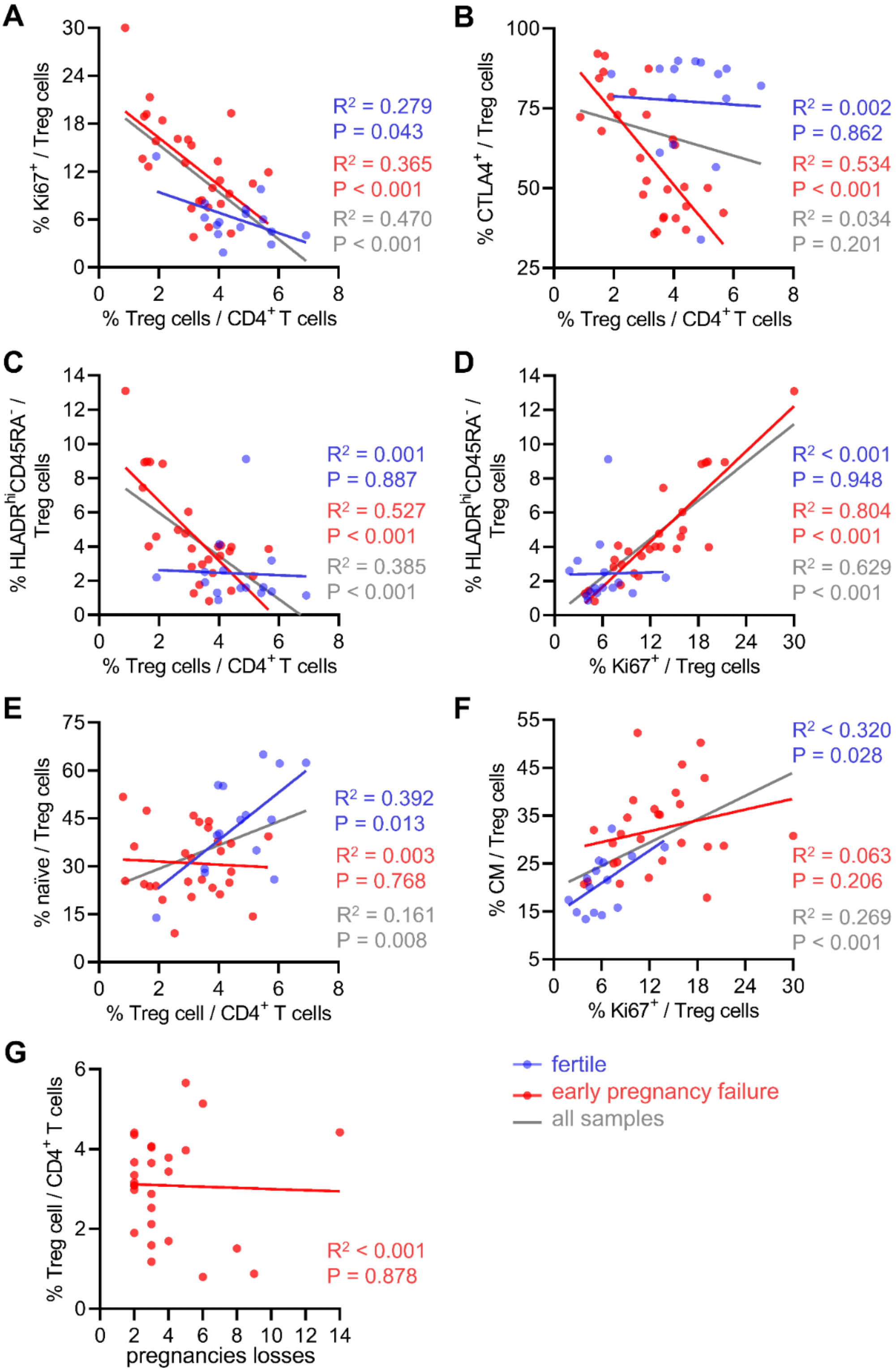

Figure S1. Relationship between Treg cell abundance and Treg cell phenotype marker expression, Related to Figure 2. Peripheral blood lymphocytes were assessed by flow cytometry from proven fertile (n = 15) and early pregnancy failure (n = 27) subjects. The % Treg cells / $CD4^{+}$ T cells was calculated, and the percentage of Treg cells expressing Ki67, CTLA4, CD45RA, CCR7 and HLADR was measured. Simple linear regression was utilized to determine if a relationship existed between the abundance of Treg cells and the proportion of Treg cells that are $Ki67^{+}$ (A); $CTLA4^{+}$ (B); $HLADR^{hi}CD45RA^{-}$ (C), or $CCR7^{+}CD45RA^{+}$ naïve Treg cells (E). In addition % $Ki67^{+}$ / Treg cells was plotted against % $HLADR^{hi}CD45RA^{-}$ / Treg cells (D) and % $CCR7^{+}CD45RA^{-}$ central memory Treg cells (F). The number of pregnancy losses in the EPF group was plotted against proportion of Treg cells (G). Data for individual proven fertile (blue) and EPF (red) subjects are shown. Relationships between all samples (grey line), fertile subjects (blue line), and EPF subjects (red line) are indicated along with the respective R^2 and *P* values.

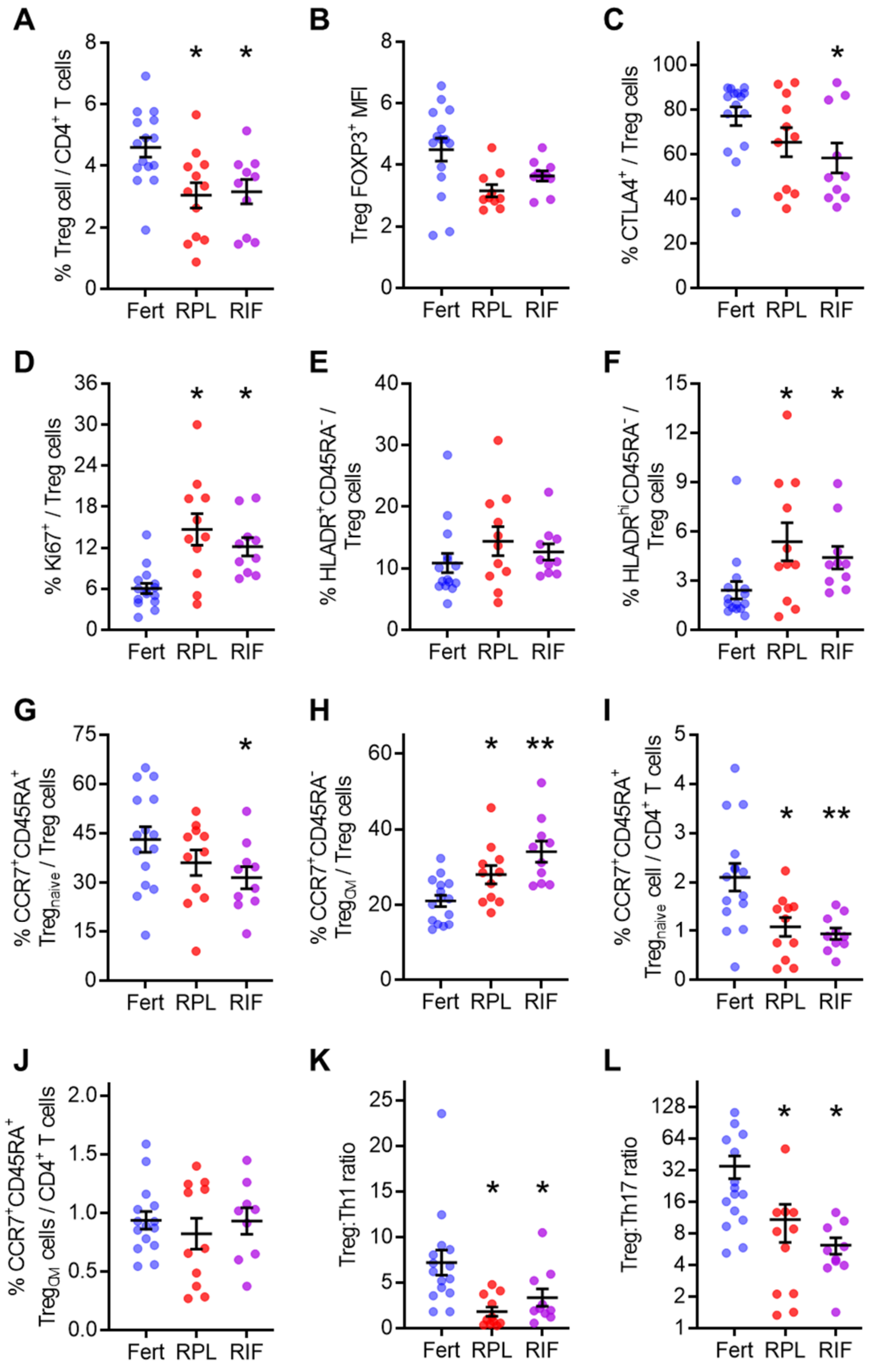

Figure S2. Peripheral blood Treg cell numbers and phenotype according to early pregnancy failure subgroup, Related to Figures 1-3. Peripheral blood lymphocytes were assessed by flow cytometry from proven fertile (Fert, n = 15) and early pregnancy failure subjects meeting diagnostic criteria for recurrent pregnancy loss (RPL, n =11) or recurrent implantation failure (RIF, n = 10). The percentage of Treg cells within $CD4^+$ T cells (A); FOXP3 MFI of Treg cells (B); $CTLA4^+$ Treg cells as a proportion of Treg cells (C); $Ki67^+$ Treg cells as a proportion of Treg cells (D); $HLADR^+CD45RA^-$ Treg cells as a proportion of Treg cells (E); $HLADR^{hi}CD45RA^-$ Treg cells as a proportion of Treg cells (F); $CCR7^+CD45RA^+$ $Treg_{naive}$ cells as a proportion of Treg cells (G), $CCR7^+CD45RA^-$ $Treg_{EM}$ cells as a proportion of Treg cells (H); $CCR7^+CD45RA^+$ $Treg_{naive}$ cells as a proportion of $CD4^+$ T cells (I), $CCR7^+CD45RA^-$ $Treg_{EM}$ cells as a proportion of $CD4^+$ T cells (J). The Treg to $CD4^+Tbet^+$ Tconv cell ratio (Treg:Th1, K) and Treg to $CD4^+ROR\gamma t^+$ Tconv cell ratio (Treg:Th17, L) were calculated. Individual study participants are shown, along with the mean±SEM of each group. Data was ANOVA and post-hoc t-test and FDR test with significant effects of subgroup are indicated by * *q*-value < 0.05, ** *q*-value < 0.01

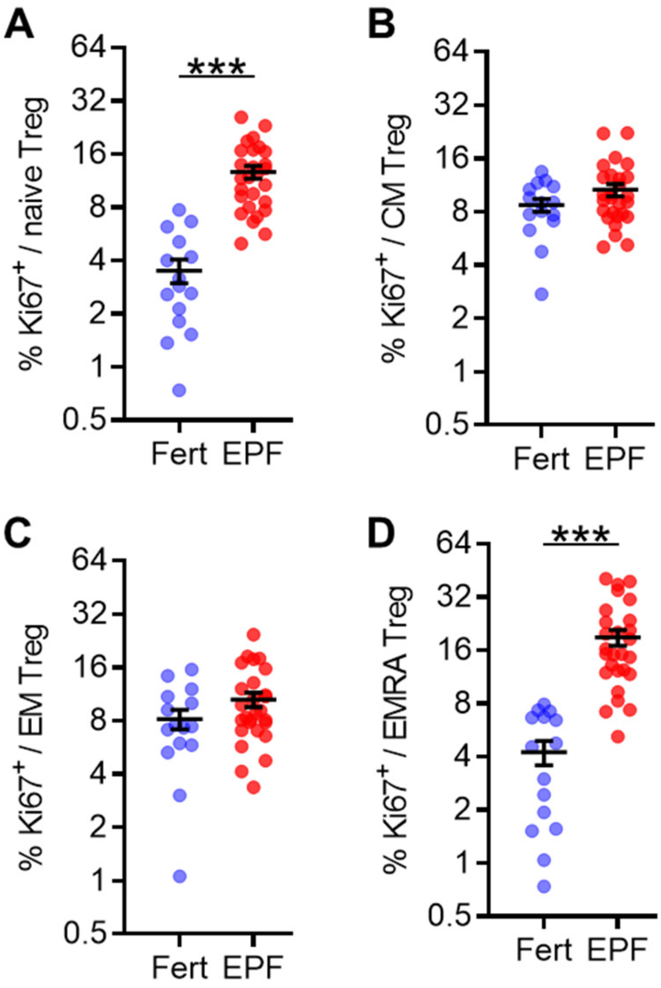

Figure S3. Relationship between fertility status and Ki67 expression in naïve and memory Treg cell subsets, Related to Figure 2. Peripheral blood lymphocytes were assessed by flow cytometry from proven fertile (Fert, n = 15) and early pregnancy loss (EPF, n = 27) subjects. $CD4^{+}FOXP3^{+}CD25^{+}CD127^{-/lo}$ Treg cells were identified and CD45RA and CCR7 expression assessed to determine naïve (A), central memory (B), effector memory (C) and terminally differentiated effector memory (D) Treg cells. The proportion of Ki67+ Treg cells within each naïve and memory subset was evaluated. Each graph shows individual study participants, and the mean±SEM of each group. Data was analyzed by t-test and FDR assessed by Benjamini, Krieger and Yekutieli test, significant effects of fertility status indicated by *** *q*-value < 0.001.

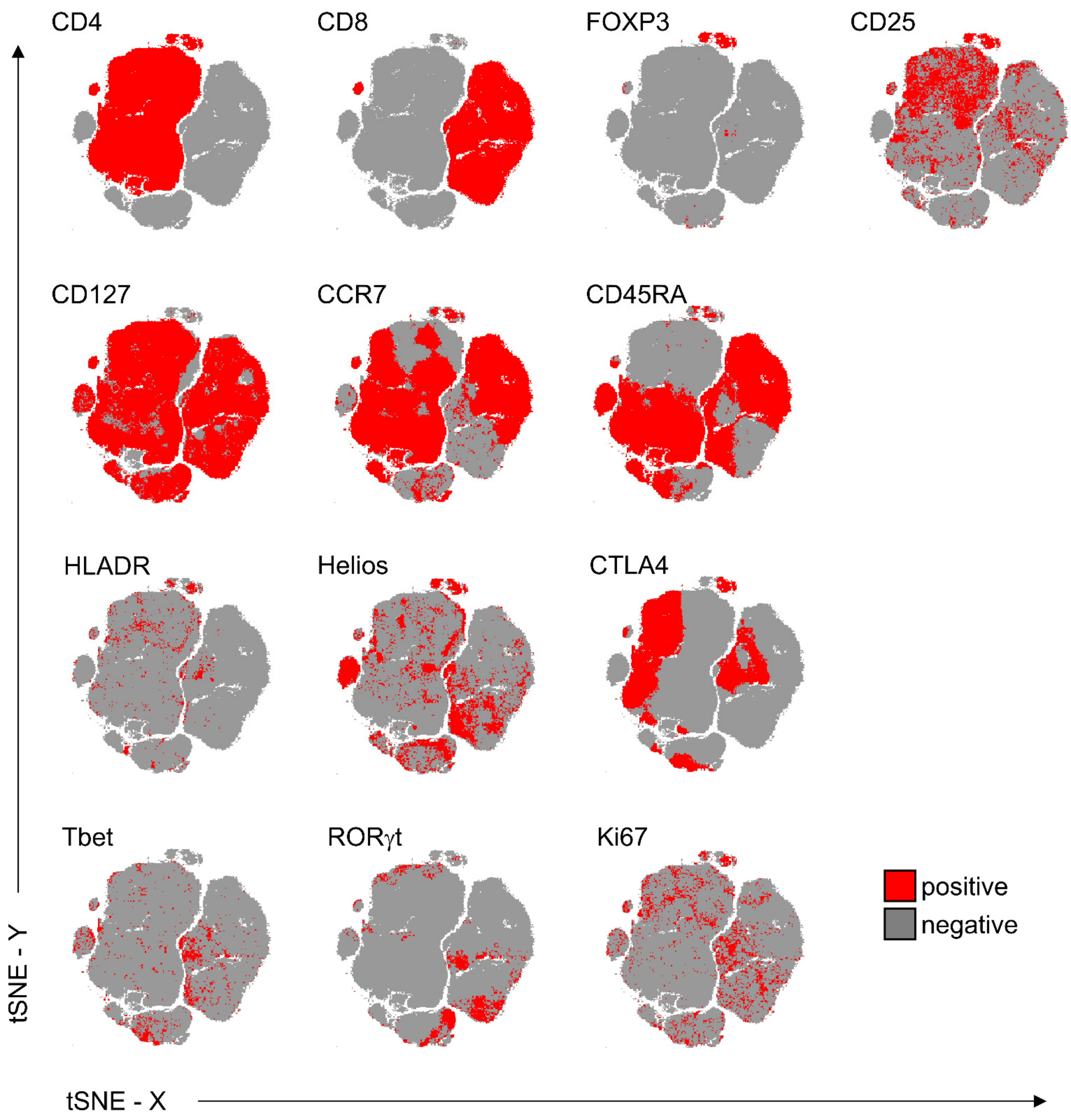

Figure S4. Marker expression in the CD3⁺ T cell tSNE, Related to Figure 4. Peripheral blood CD3⁺ T cells from proven fertile (n = 15) and early pregnancy failure subjects (n = 27) were assessed by flow cytometry and a down sampling of cells from each sample was concatenated into a single file that was visualised using the tSNE algorithm. Manuel gates were established to capture cells that were positive for CD4, CD8, FOXP3, CD25, CD127, CCR7, CD45RA, HLADR, Helios, CTLA4, Tbet, RORγt or Ki67, and these positive cells were mapped to the tSNE plot and indicated by red colouring.

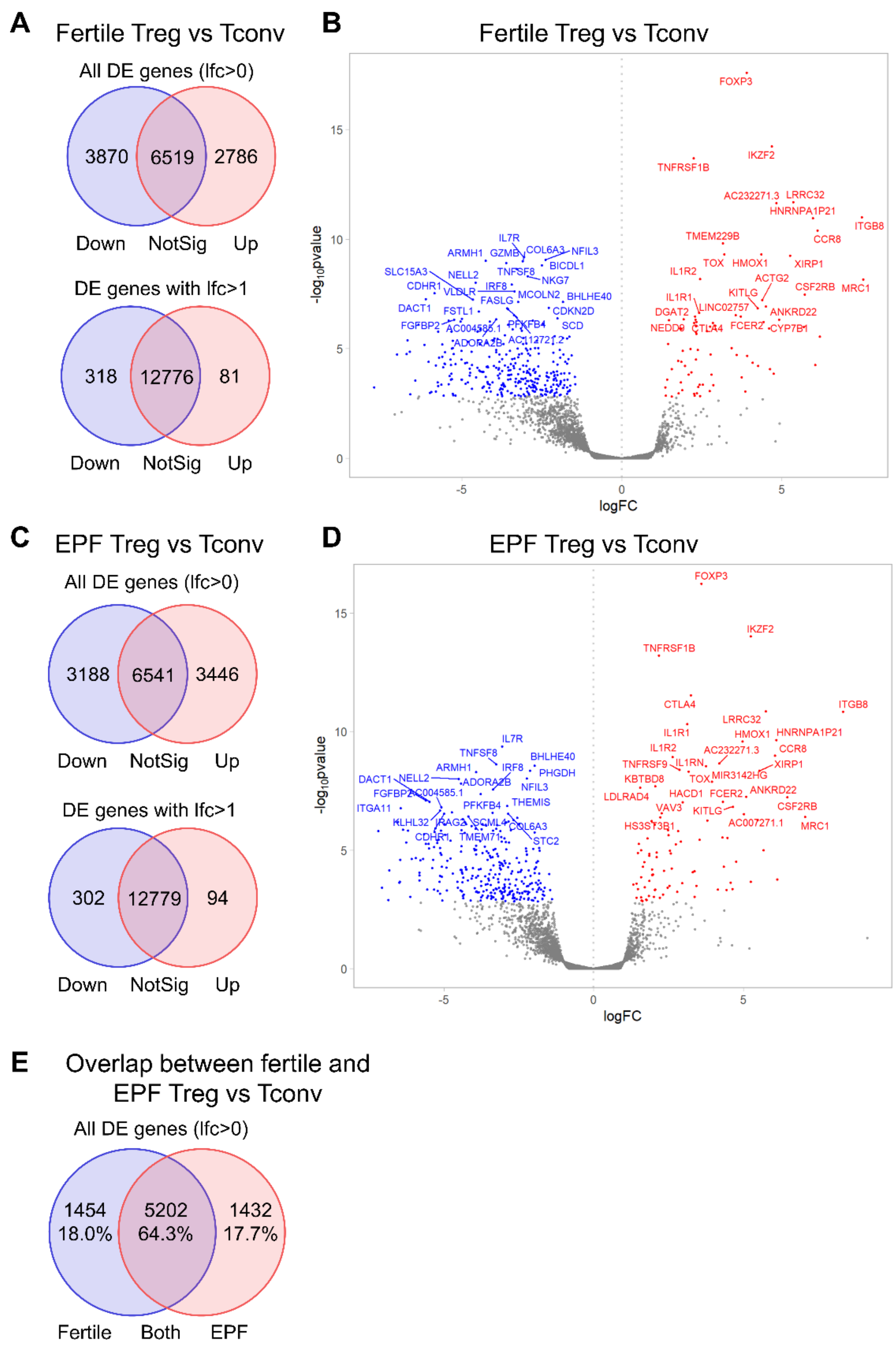

Figure S5. Differentially expressed genes in Treg cells versus Tconv cells within early pregnancy failure and proven fertile control groups, Related to Figure 6. Differentially expressed genes identified in Treg cells (n = 6) versus Tconv cells (n = 11) of fertile subjects with either no log-fold change (lfc) cut-off, or with a lfc > 1 threshold equivalent to a 2-fold difference in level of expression, is shown (A). A volcano plot of differentially expressed genes in Treg cells versus Tconv cells from fertile subjects shows the top 50 most significantly differentially expressed genes, with upregulated genes in Treg cells coloured red and downregulated genes coloured blue (B). Differentially expressed genes identified in Treg cells (n = 8) versus Tconv cells (n = 12) of EPF subjects with either no log-fold change (lfc) cut-off, or with a lfc > 1 threshold equivalent to a 2-fold difference in level of expression (C). A volcano plot of differentially expressed genes in Treg cells versus Tconv cells of fertile subjects was constructed with upregulated genes in Treg cells indicated by red and downregulated genes shown in blue (D). Overlap in Treg versus Tconv cells differentially expressed genes identified in fertile compared with EPF subjects. The number of genes is given together with proportion represented from the total differentially expressed genes in the two comparative sets (E). An FDR $P < 0.05$ cut-off was used to define statistical significance for gene sets.

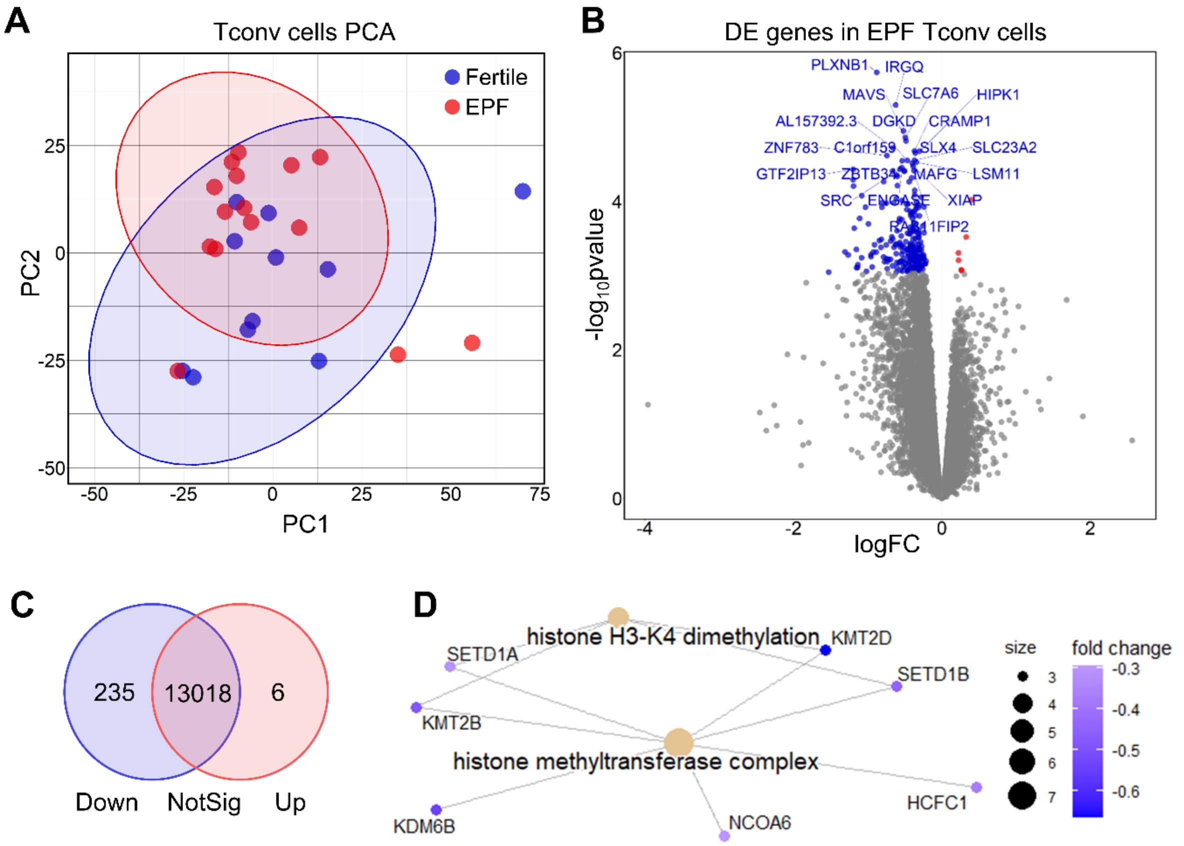

Figure S6. Differentially expressed genes and enriched pathways in Tconv cells from early pregnancy loss subjects versus proven fertile controls, Related to Figure 6. A PCA plot shows moderate clustering of Tconv cell samples by fertility status with ellipses marking the 95% CI for the proven fertile (n = 11, blue) or EPF (n=15, red) samples (A). Prior to analysis of differentially expressed genes, 3 samples (red) lying outside the 95% CI for the RPL group were excluded and the new PCA plot is shown in Figure 6C. A volcano plot shows differentially expressed genes in Tconv cells from EPF subjects (n = 12) versus proven fertile subjects (n = 11) (B). The top 20 genes are labelled. Significantly upregulated genes (FDR $P < 0.05$) are red, and downregulated genes are blue. The number of differentially expressed genes in Tconv cells from EPF versus proven fertile subjects for pathway analysis, after applying a log-fold change threshold of 0.04, equivalent to a 1.03-fold difference in level of expression, shown as a Venn diagram (C). A GO network plot for all enriched GO terms associated with differentially expressed genes identified in Tconv cells from EPF versus proven fertile subjects (D).

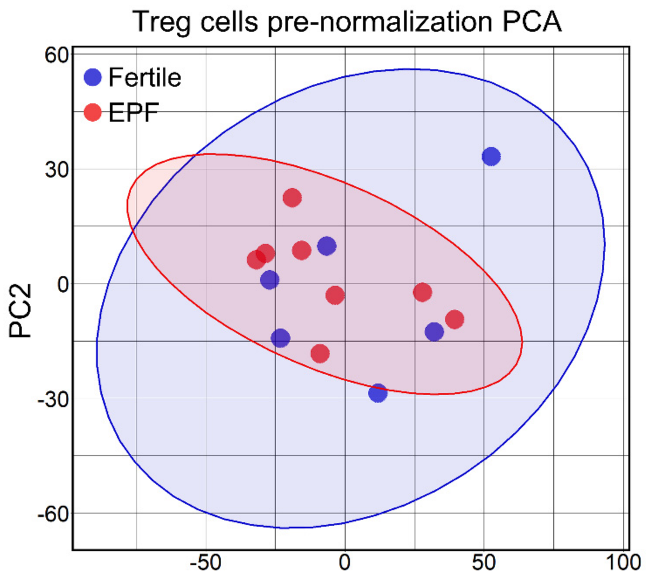

Figure S7. PCA of Treg cell samples without RUVg normalization, Related to Figure 6. A PCA plot of RNAseq samples of Treg cells from early pregnancy failure (EPF) versus proven fertile (Fert) subjects before RUVg normalization. The ellipses indicate the 95% CI for clustering around the mean for fertile (n= 6, blue) or EPF (n=8, red) groups.

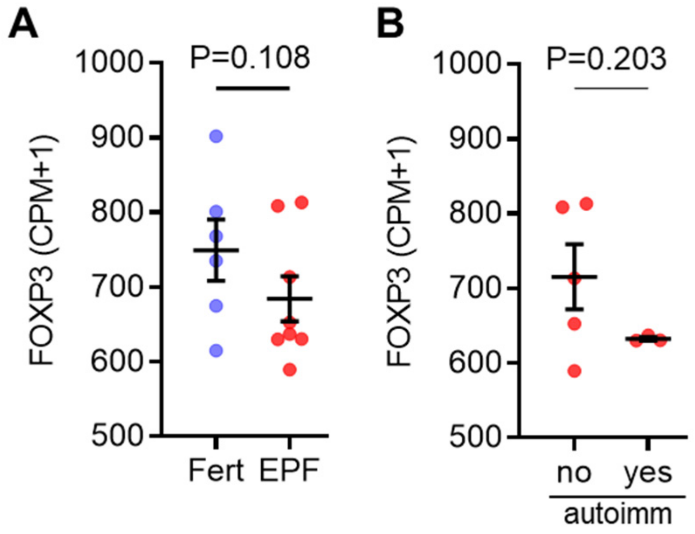

Figure S8. Relationship between FOXP3 expression and fertility status or autoimmune status, Related to Figure 7. Expression of *FOXP3* in Treg cells from fertile (Fert) or early pregnancy failure (EPF) subjects by RNAseq was analysed by one-tailed unpaired t-test (A). Subjects within the EPF group were assigned status of 'no' or 'yes' for evidence of autoimmune dysfunction based upon testing positive for antinuclear, anticardipliin, B2-glycoprotein antibodies, lupus anticoagulant or altered thyroid stimulating hormone as per the ESHRE guidelines for testing RPL patients (see Table S2). Expression of *FOXP3* in Treg cells from EPF subjects by RNAseq was plotted according to autoimmunity status (B).

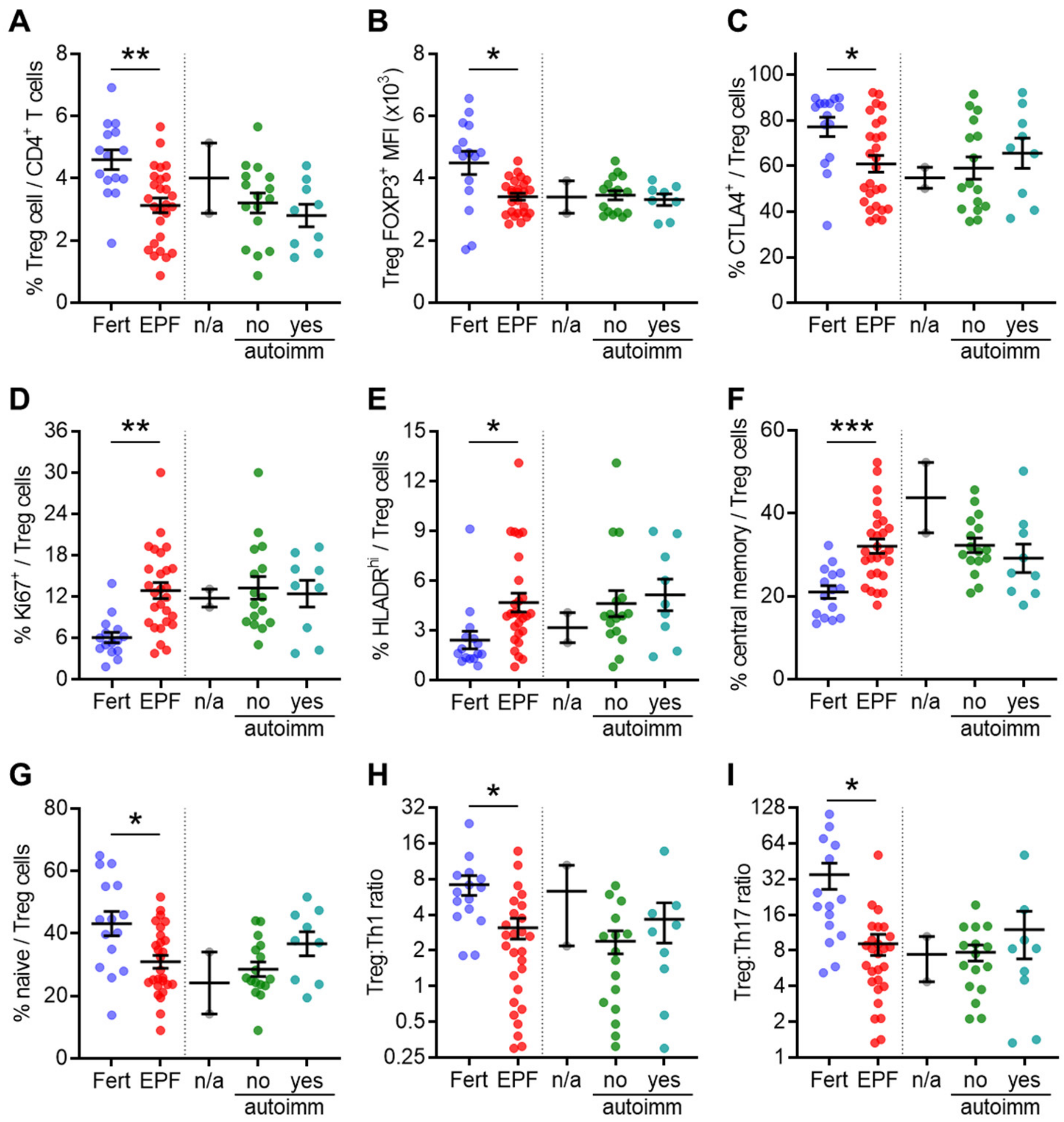

Figure S9. Relationship between flow cytometry Treg cell parameters and autoimmune status, Related to Figure 8. Peripheral blood CD4^{+} T cells were assessed by flow cytometry in proven fertile (Fert, n = 15) and early pregnancy failure patients (EPF, n = 27). EPF patients were assigned status of 'no' or 'yes' for evidence of autoimmune dysfunction based upon testing positive for antinuclear, anticardipliin, B2-glycoprotein antibodies, lupus anticoagulant or altered thyroid stimulating hormone as per the ESHRE guidelines for testing RPL patients (see Table S2). Two patients did not have blood test results available (n/a). The proportion of Treg cells, defined as FOXP3^{+}CD25^{+}CD127$^{-/lo}$ cells within the CD3^{+}CD4^{+} T cells was measured (A). Within the Treg cell population the extent of FOXP3 expression, as measured by MFI, was assessed (B). The proportion of Treg cells positive for CTLA4 (C), Ki67 (D) and HLADR^{hi} (E) were calculated. The percentage of CCR7^{+}CD45RA^{-} central memory Treg (Treg$_{CM}$) cells (F) and CCR7^{+}CD45RA^{+} Treg$_{naïve}$ cells (G) within the Treg cell population were measured. Th1 cells within the Tconv population were defined by Tbet expression and the Treg:Th1 ratio calculated (H). Th17 cells within the Tconv population were defined by RORγt expression and the Treg:Th17 ratio calculated (I). Symbols indicate individual study participants, the mean±SEM of each group are shown. To determine differences between fertility status, Fert and EPF subjects were analyzed by t-test with FDR test, significant differences indicated by * *q*-value < 0.05, ** *q*-value < 0.01, *** *q*-value < 0.001. To determine if autoimmune status impacted Treg cell parameters within EPF subjects, the no autoimm and yes autoimm groups were compared by t-test with FDR correction, no differences were detected.

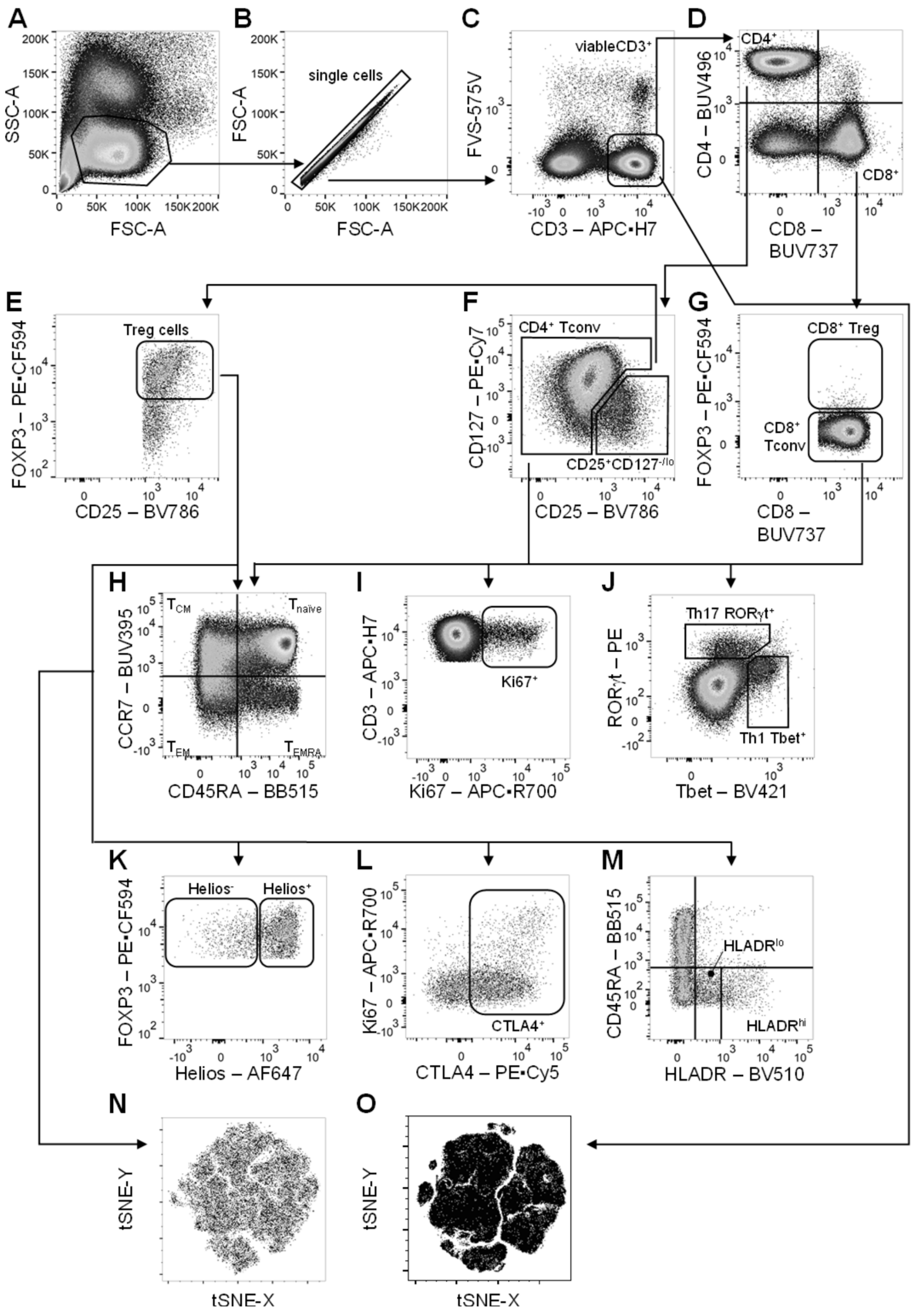

Figure S10. Gating strategy for analysis of T cell subsets, Related to Figures 1-5. Gates were established to include lymphocytes and exclude debris and doublets, based on forward (FSC) and side (SSC) scatter profiles (A, B). Cells were then applied to a CD3 versus fixable viability dye plot to identify viable CD3$^+$ cells (C) from which CD4$^+$, CD8$^+$, CD4$^+$CD8$^+$, and CD4$^-$CD8$^-$ T cells were identified (D). Within CD4$^+$ T cells, CD25, CD127, and FOXP3 were utilized to gate both CD4$^+$ Tconv and CD4$^+$CD25$^+$CD127$^{-/lo}$FOXP3$^+$ Treg cells (E, F). CD8$^+$FOXP3$^-$ Tconv and CD8$^+$FOXP3$^+$ Treg cells were identified (G). CD4$^+$ Treg, CD4$^+$ Tconv, and CD8$^+$ Tconv cells were assessed for their memory and naïve status using CCR7 and CD45RA expression, with naïve ($T_{naïve}$), central memory (T_{CM}), effector memory (T_{EM}) and effector memory expressing CD45RA (T_{EMRA}) cells identified (H). Proliferation was assessed by Ki67 expression in the Tconv and Treg cell compartments (I) and Th1 and Th17 phenotypes were examined by measuring the transcription factors Tbet and RORγt, respectively (J). Within the CD4$^+$ Treg cell population the expression of key suppression and stability markers Helios (K) and CTLA4 (L) were assessed. Treg cell suppressive phenotype was analyzed by measuring the proportion of Treg cells that were CD45RA$^+$HLADR$^-$, CD45RA$^-$HLADR$^-$, CD45RA$^-$HLADR$^+$, CD45RA$^-$HLADR^{lo} and CD45RA$^-$HLADR^{hi} (M). Treg cell tSNE analysis (N) was performed on the CD3$^+$CD4$^+$CD25$^+$CD127$^{-/lo}$FOXP3$^+$ Treg cells identified in Figure E. Whole T cell tSNE analysis (O) was conducted on the CD3$^+$ population gated in Figure C. RNAseq was performed on two cell subsets which were sorted by a BD FACS Aria Fusion, a Treg cell population, defined as CD25$^+$CD127$^{-/lo}$, and a Tconv cell population, both of which are gated in panel F.

Table S1. Clinical information for study participants, by group, Related to Figures 1-8.

	Proven Fertile	Early Pregnancy Failure	Recurrent Pregnancy Loss[a]	Recurrent Implantation Failure
number	15	27	11	10
age (yrs)[b, c]	34.8 ± 1.7<br>(26.9 - 45.0)	36.3 ± 0.8<br>(28.0 - 44.1)	34.8 ± 1.0<br>(30.3-39.5)	36.7 ± 1.3<br>(32.2 - 42.9)
BMI	23.7 ± 0.9<br>(19.7 - 29.4)	24.1 ± 0.8<br>(19.2 – 35.1)	23.6 ± 0.8<br>(19.2 - 25.7)	22.8 ± 0.5<br>(20.8 - 25.1)
clinical pregnancies	1.7 ± 0.2<br>(1 - 4)	2.5 ± 0.5<br>(0 - 12)	4.5 ± 0.9 *<br>(2 - 12)	1.7 ± 0.8<br>(0 - 8)
live births	1.7 ± 0.2<br>(1 - 4)	0.4 ± 0.1 *<br>(0 - 2)	0.5 ± 0.2 *<br>(0 - 2)	0.4 ± 0.2 *<br>(0 - 2)
pregnancy losses (miscarriages)	0.1 ± 0.1<br>(0 - 1)	1.9 ± 0.4 *<br>(0 - 9)	3.7 ± 0.7 *<br>(2 - 9)	1.0 ± 0.6<br>(0 - 6)
implantation failures	0.0 ± 0.0<br>(0 - 0)	2.1 ± 0.4 *<br>(0 - 8)	1.3 ± 0.8<br>(0 - 8)	4.4 ± 0.6 *<br>(3 - 8)
early pregnancy failures[d]	0.1 ± 0.1<br>(0 - 1)	4.0 ± 0.5 *<br>(2 - 14)	5.0 ± 1.1 *<br>(2 - 14)	5.4 ± 1.1 *<br>(3 - 14)
elective terminations	0.0 ± 0.0<br>(0 - 0)	0.1 ± 0.1<br>(0 - 2)	0.0 ± 0.0<br>(0 - 0)	0.2 ± 0.2<br>(0 - 2)
ectopic pregnancies	0.0 ± 0.0<br>(0 - 0)	0.04 ± 0.04<br>(0 - 1)	0.1 ± 0.1<br>(0 - 1)	0.0 ± 0.0<br>(0 - 0)
stillbirths	0.0 ± 0.0<br>(0 - 0)	0.1 ± 0.1<br>(0 - 1)	0.1 ± 0.1<br>(0 - 1)	0.1 ± 0.1<br>(0 - 1)
total pregnancy failures[e]	0.1 ± 0.1<br>(0 - 1)	4.2 ± 0.6 *<br>(2 - 15)	5.2 ± 1.2 *<br>(2 - 15)	5.7 ± 1.2 *<br>(3 - 15)

[a] Subsets of subjects with early pregnancy failure (EPF) were further classified as recurrent pregnancy loss (RPL) or recurrent implantation failure (RIF) when clinical criteria for these conditions were met (see Materials and Methods).
[b] All participant parameters are shown as mean±SEM and range.
[c] Differences between proven fertile subjects and EPF, RPL, or RIF groups were evaluated by ANOVA and Dunnett's t test. * $P < 0.05$ compared to fertile group.
[d] total early pregnancy failures includes miscarriages and implantation failure.
[e] total losses includes miscarriages, implantation failure, stillbirth and elective terminations, including ectopic pregnancies.

Table S2. Clinical information for individual study participants, Related to Figures 1-8.

Subject	RPL / RIF	Clin preg	Live births	Misc.	IVF implant failures	Other preg losses	Autoantibody parameters[a]	Autoimmune status[e]
Fert 1 ^*		2	2	-	-	-	n/a	n/a
Fert 2 ^		4	4	-	-	-	n/a	n/a
Fert 3 ^*		2	2	-	-	-	n/a	n/a
Fert 4		2	2	-	-	-	n/a	n/a
Fert 5 ^		1	1	-	-	-	n/a	n/a
Fert 6 ^		2	2	-	-	-	n/a	n/a
Fert 7 ^*		3	3	-	-	-	n/a	n/a
Fert 8 ^		1	1	-	-	-	n/a	n/a
Fert 9 ^*		2	2	-	-	-	n/a	n/a
Fert 10 ^*		2	1	1	-	-	n/a	n/a
Fert 11		1	1	-	-	-	n/a	n/a
Fert 12 ^		1	1	-	-	-	n/a	n/a
Fert 13		1	1	-	-	-	n/a	n/a
Fert 14		1	1	-	-	-	n/a	n/a
Fert 15 ^*		1	1	-	-	-	n/a	n/a
EPF 1 ^*	RIF	-	-	-	4	-	all negative	No
EPF 2 ^*	RIF	-	-	-	3	-	ANA+	Yes
EPF 3	RIF	-	-	-	3	-	all negative	No
EPF 4 ^	RIF	4	2	-	3	-	all negative	No
EPF 5	RIF	1	-	1	5	-	n/a	n/a
EPF 6 ^	RIF	-	-	-	3	-	n/a	n/a
EPF 7	RIF	1	1	-	4	-	all negative	No
EPF 8 ^	RIF	1	0	1	7	-	all negative	No
EPF 9 ^*	RPL/RIF	8	1	6	8	1 stillbirth	all negative	No
EPF 10	RPL/RIF	2	0	2	4	-	ANA+++	Yes
EPF 11	RPL	2	-	2	-	-	all negative	No
EPF 12	RPL	2	-	2	-	-	ANA++	Yes
EPF 13 ^*	RPL	5	-	5	-	-	all negative	No
EPF 14	RPL	5	2	3	2	-	ANA+	Yes
EPF 15 ^	RPL	3	-	3	-	-	ANA+++	Yes
EPF 16 ^*	RPL	4	-	4	-	-	all negative	No
EPF 17	RPL	4	1	3	-	-	all negative	No
EPF 18	RPL	12	2	9	-	1 ectopic	all negative	No
EPF 19	RPL	2	-	2	-	-	all negative	No
EPF 20		1	-	1	2	-	all negative	No
EPF 21		1	-	1	1	-	all negative	No
EPF 22 ^		1	-	1	1	-	LA+	Yes
EPF 23		1	-	1	1	-	all negative	No
EPF 24 *		2	1	1	1	-	all negative	No
EPF 25		2	1	-	2	1 termin.	ANA+	Yes
EPF 26 ^*		2	-	1	2	1 stillbirth	TSH+	Yes
EPF 27 ^*		1	-	1	1	-	ANA++, HT	Yes

^*Samples where CD4 Tconv cells (^) and/or Treg cells (*) were analysed by RNAseq are indicated. [a]Autoantibody parameters (titres of antinuclear antibody, ANA; anticardiolipin, ACA; B2-glycoprotein, B2GP; lupus anticoagulant, LA, and thyroid stimulating hormone) were available for most early pregnancy failure (EPF) subjects (25/27) but were not available for proven fertile (Fert) subjects. ANA+ = 1:80; ANA++ = 1:160; ANA+++ = 1:640; LA+ = positive, TSH+ = > 4.5 mIU/L, HT = Hashimoto's thyroiditis. Autoimmune status was designated 'Yes' or 'No' according to ESHRE guidelines recommending testing α-nuclear Ab, α-phospholipid Abs, and thyroid function. Abbreviations: n/a = not available. Clin preg = clinical pregnancies, misc. = miscarriages, termin. = termination.

Table S3. Differentially expressed genes in Tconv cells from early pregnancy failure subjects compared with Tconv cells from fertile subjects, Related to Figure 6.

See Supplemental File *Table S3.xls*

Table S4. Gene Set Enrichment Analysis results for differentially expressed genes in Tconv cells of early pregnancy failure subjects, Related to Figure 6.

Collection	Pathway	ES[a]	NES	Size	Adj*P*
C2 - CGP	GINESTIER_BREAST_CANCER_20Q13_ AMPLIFICATION_DN	-0.6153	-1.8021	166	1.08E-08
C2 - CGP	LAIHO_COLORECTAL_CANCER_ SERRATED_DN	-0.67366	-1.8928	76	4.03E-05
C2 - CGP	BILANGES_SERUM_RESPONSE _TRANSLATION	0.82802	2.6569 2	19	0.000425
C2 - CGP	TIEN_INTESTINE_PROBIOTICS_6HR_UP	0.59509 6	2.3756 5	48	0.00054
C2 - CGP	MENSE_HYPOXIA_UP	0.47208 8	2.1318 8	90	0.000841
C2 - CGP	FARDIN_HYPOXIA_11	0.68714 9	2.5150 2	31	0.001601
C2 - CGP	ZHOU_CELL_CYCLE_GENES_IN_IR_ RESPONSE_6HR	0.48846 5	2.1042 9	78	0.001734
C2 - CGP	BURTON_ADIPOGENESIS_3	0.45155 3	2.0244 6	89	0.002538
C2 - CGP	BHATTACHARYA_EMBRYONIC_STEM_CE LL	0.51987 2	2.0612 8	64	0.003868
C2 - CGP	FLOTHO_PEDIATRIC_ALL_THERAPY_ RESPONSE_UP	0.56101 7	2.2396 1	48	0.00684
C2 - CGP	KONG_E2F3_TARGETS	0.44016 6	2.0793 5	87	0.023178
C2 - CGP	BILANGES_SERUM_AND_RAPAMYCIN_ SENSITIVE_GENES	0.55271 1	2.1861 9	46	0.037571
C2 - CP:KEGG	KEGG_RIBOSOME	0.64733 4	2.8788 0	84	4.75E-11
C2 - CP:REACTOME	REACTOME_ACTIVATION_OF_THE_MRNA _ UPON_BINDING_OF_THE_CAP_BINDING_ COMPLEX_AND_EIFS_AND _SUBSEQUENT _BINDING_TO_43S	0.55998 8	2.2629 5	58	0.000726
C2 - CP:REACTOME	REACTOME_SLC_MEDIATED _TRANSMEMBRANE_TRANSPORT	-0.58094	-1.6808	123	0.002265
C3 - MIR:MIR_Legacy	GAGCTGG_MIR337	-0.59318	-1.7189	128	0.000179
C3 - MIR:MIR_Legacy	CTGAGCC_MIR24	-0.53163	-1.5586	176	0.014206
C3 - MIR:MIR_Legacy	CACTGCC_MIR34A_MIR34C_MIR449	-0.51039	-1.5044	215	0.018025
C3 - MIR:MIR_Legacy	GTACTGT_MIR101	-0.51314	-1.5124	204	0.025025
C7 - IMMUNESIGDB	GSE13485_DAY1_VS_DAY21_YF17D _VACCINE_PBMC_UP	-0.59302	-1.7342	156	3.19E-06
C7 - IMMUNESIGDB	GSE45837_WT_VS_GFI1_KO_PDC_UP	-0.62721	-1.8077	113	1.18E-05
C7 - IMMUNESIGDB	GSE37532_VISCERAL_ADIPOSE_TISSUE _VS_LN_DERIVED_PPARG_KO_TREG _CD4_TCELL_DN	-0.60563	-1.7485	116	9.95E-05
C7 - IMMUNESIGDB	GSE2405_S_AUREUS_VS_A _PHAGOCYTOPHILUM_NEUTROPHIL_DN	-0.55204	-1.6195	181	0.000374

C7 - IMMUNESIGDB	GSE45881_CXCR6HI_VS_CXCR1LO _COLONIC_LAMINA_PROPRIA_UP	-0.54458	-1.5994	186	0.000486
C7 - IMMUNESIGDB	GSE16385_MONOCYTE_VS_12H_IL4 _TREATED_MACROPHAGE_DN	-0.54359	-1.5965	187	0.000774
C7 - IMMUNESIGDB	GSE7509_UNSTIM_VS_FCGRIIB _STIM_MONOCYTE_DN	-0.55362	-1.6201	159	0.003085
C7 - IMMUNESIGDB	GSE27241_WT_VS_RORGT_KO_TH17 _POLARIZED_CD4_TCELL_TREATED _WITH_DIGOXIN_UP	-0.54354	-1.5910	168	0.003689
C7 - IMMUNESIGDB	GSE19888_CTRL_VS_A3R _ACTIVATION_MAST_CELL_UP	-0.54831	-1.6049	158	0.007415
C7 - IMMUNESIGDB	GSE23925_DARK_ZONE_VS _NAIVE_BCELL_DN	-0.54391	-1.5921	162	0.008109
C7 - IMMUNESIGDB	GSE29617_DAY3_VS_DAY7_TIV _FLU_VACCINE_PBMC_2008_DN	-0.55517	-1.6159	141	0.010731
C7 - IMMUNESIGDB	GSE17301_CTRL_VS_48H_IFNA2 _STIM_CD8_TCELL_DN	-0.54381	-1.5894	153	0.012476
C7 - IMMUNESIGDB	GSE13485_DAY3_VS_DAY21_YF17D _VACCINE_PBMC_UP	-0.52876	-1.5502	176	0.018043
C7 - IMMUNESIGDB	GSE19772_CTRL_VS_HCMV_INF _MONOCYTES_AND_PI3K_INHIBITION_UP	-0.54569	-1.5906	144	0.026451
C7 - IMMUNESIGDB	GSE13493_DP_VS_CD4INTCD8POS _THYMOCYTE_UP	-0.52833	-1.5474	166	0.036968

[a]ES, enrichment score; NES, normalized enrichment score (normalized to mean enrichment of random samples of the same size); Adj*P*, Bonferroni-adjusted *P* value; CP, canonical pathways; CGP, chemical and genetic perturbations; PID, Pathway Interaction Database.

Table S5. Differentially expressed genes in Treg cells of early pregnancy failure subjects compared to Treg cells of fertile subjects, Related to Figure 6.

See Supplemental File *Table S5.xls*

Table S6. Enriched GO terms for differentially expressed genes in Treg cells of early pregnancy failure subjects, Related to Figure 6.

Description	GeneRatio	BgRatio[a]	Adj*P*	FDR	GeneID
myeloid leukocyte migration	13/168	126/10717	0.00026	0.00021	*CCL3, CCL4, IL17A, CCL20, IL4, MCOLN2, MTUS1, CD200, RIN3, PTPRO, PTGER4, FLT1, CD9*
regulation of cell migration	24/168	547/10717	0.01401	0.00214	*CLDN1, FAM110C, PTGER4, CCL3, CCL4, IL24, IFNG, CD9, CCL20, IL4, PTGS2, PTPRU, SMPD3, MTUS1, CD200, NOG, RIN3, KLF4, CD151, SEMA7A, ADGRB1, NTN1, FLT1, DNAI3*
regulation of locomotion	25/168	594/10717	0.01806	0.00214	*CCL3, CCL4, IL24, IFNG, CCL20, IL4, PTGS2, CD9, PTPRU, SMPD3, MTUS1, CD200, NOG, RIN3, KLF4, CD151, SEMA7A, ADGRB1, CLDN1, FAM110C, PTPRO, PTGER4, NTN1, FLT1, DNAI3*
regulation of cytokine production	22/168	486/10717	0.02233	0.00214	*CSF2, CCL3, IFNG, IL17A, IL21, IL4, IL10, MCOLN2, PTGS2, CD200, LPL, KLF4, TNFSF4, SEMA7A, CD40LG, ARRB1, POMC, PTGER4, UBASH3A, IRF7, IL9, BTN3A3*
hematopoietic or lymphoid organ development	26/168	643/10717	0.02363	0.00214	*CSF2, CCL3, IFNG, IL17A, IL21, IL4, IL10, IL3, ITM2A, CXCR5, SMPD3, SOX13, KLF4, TNFSF4, MB, TCF7, CD40LG, CTLA4, ZFAT, PTGER4, NFIL3, PLEK, THEMIS, FLT1, IRF7, IL9*
cytokine production	22/168	496/10717	0.03084	0.00214	*CSF2, CCL3, IFNG, IL17A, IL21, IL4, IL10, MCOLN2, PTGS2, CD200, LPL, KLF4, TNFSF4, SEMA7A, CD40LG, ARRB1, POMC, PTGER4, UBASH3A, IRF7, IL9, BTN3A3*
positive regulation of tyrosine phosphorylation of STAT protein	6/168	33/10717	0.03412	0.00214	*CSF2, IL24, IFNG, IL21, IL4, IL3*
G protein-coupled receptor signaling pathway	17/168	322/10717	0.03776	0.00214	*CCL3, CCL4, CCL20, CXCR5, HPGD, ADGRB1, DGKI, RGS6, ARRB1, OMC, PTGER4, PLEK, GPR65, GPR160, RGS1, PRKACB, GPR146*

integral component of plasma membrane	27/177	645/11166	0.00112	0.00101	*SEMA7A, ADGRB1, CSF2, SLC7A8, CXCR5, PTPRU, CD200, TRPM6, CALHM6, CD151, TNFSF4, CLDN1, CD40LG, CTLA4, SLCO4C1, KCNC3, TSPAN33, PTPRO, B3GNT3, SLC25A4, GPR65, EDA, IL17RB, FLT1, CD9, CHRNA6, P2RX5*
plasma membrane region	24/177	630/11166	0.01934	0.00581	*SLC4A10, SLC7A8, CLIC5, SORBS1, PTGS2, HPGD, TRPM6, EHD2, SLC4A8, CLDN1, DGKI, OCEL1, SLCO4C1, KCNC3, ARRB1, NHS, PTPRO, PTCH1, ANK3, PLEK, NAALADL1, CHRNA6, TGFA, LMO7*
apical part of cell	12/177	203/11166	0.03196	0.00720	*SLC4A10, SLC7A8, CLIC5, TRPM6, CLDN1, OCEL1, NHS, PTPRO, PTCH1, EDA, NAALADL1, LMO7*
cell surface	18/177	422/11166	0.04400	0.00793	*IL17A, SDC2, CXCR5, CD200, LPL, PDCD1, CD151, TNFSF4, SEMA7A, CD40LG, CTLA4, TSPAN33, TNS1, ANK3, CD9, BTN3A3, TGFA, LMO7*
signaling receptor regulator activity	23/180	171/11069	1.88E-12	1.61E-12	*CSF2, CCL3, CCL4, IGF2, IL24, IFNG, IL17A, CCL20, IL21, IL4, IL10, ESR2, IL3, TNFSF4, UTS2, SEMA7A, CD40LG, METRNL, POMC, EDA, IL9, TGFA, IL31*
receptor ligand activity	22/180	159/11069	4.23E-12	1.81E-12	*CSF2, CCL3, CCL4, IGF2, IL24, IFNG, IL17A, CCL20, IL21, IL4, IL10, IL3, TNFSF4, UTS2, SEMA7A, CD40LG, METRNL, POMC, EDA, IL9, TGFA, IL31*
cytokine receptor binding	14/180	149/11069	5.94E-05	1.02E-05	*CSF2, CCL3, CCL4, IFNG, CCL20, IL21, IL4, IL10, IL3, EDA, TNFSF4, CD40LG, IL9, IL31*

[a]BgRatio, background ratio; Adj*P*, Bonferroni-adjusted *P* value, FDR, false discovery rate *P* value (*q* value).

Table S7. Enriched Kegg terms for differentially expressed genes in Treg cells of early pregnancy failure subjects, Related to Figure 6.

Kegg ID	Description	GeneRatio	BgRatio[a]	Adj*P*	FDR	geneID
hsa04060	Cytokine-cytokine receptor interaction	18/85	147/5026	4.23E-09	3.83E-09	*CSF2, CCL3, CCL4, IL24, IFNG, IL17A, CCL20, IL21, IL4, IL10, IL3, CXCR5, TNFSF4, CD40LG, EDA, IL17RB, IL9, IL31*
hsa05310	Asthma	5/85	20/5026	0.00337	0.00153	*IL4, IL10, IL3, CD40LG, IL9*
hsa04630	JAK-STAT signaling pathway	9/85	97/5026	0.00677	0.00205	*CSF2, IL24, IFNG, IL21, IL4, IL10, IL3, IRF9, IL9*
hsa04657	IL-17 signaling pathway	7/85	62/5026	0.01544	0.00344	*CSF2, IFNG, IL17A, CCL20, IL4, PTGS32, IL17RB*
hsa05323	Rheumatoid arthritis	7/85	64/5026	0.01898	0.00344	*CSF2, CCL3, IFNG, IL17A, CCL20, CTLA4, FLT1*

[a]BgRatio, background ratio; Adj*P*, Bonferroni-adjusted *P* value; FDR, false discovery rate *P* value (*q* value).

Table S8. Gene set enrichment analysis results for differentially expressed genes in Treg cells of early pregnancy failure subjects, Related to Figure 6.

collection	pathway	ES[a]	NES	size	Adj*P*
H	HALLMARK_E2F_TARGETS	-0.54513	-2.12388	199	6.72E-07
H	HALLMARK_TNFA_SIGNALING_VIA_NFKB	0.61062	2.06528	168	6.02E-05
H	HALLMARK_G2M_CHECKPOINT	-0.50156	-1.95593	191	0.00043
C2 - CGP	FLORIO_NEOCORTEX_BASAL _RADIAL_GLIA_DN	-0.62670	-2.41156	154	1.99E-09
C2 - CGP	ROSTY_CERVICAL_CANCER _PROLIFERATION_CLUSTER	-0.63188	-2.37718	139	7.05E-09
C2 - CGP	PUJANA_XPRSS_INT_NETWORK	-0.56057	-2.15684	168	1.08E-06
C2 - CGP	PUJANA_BRCA_CENTERED_NETWORK	-0.61473	-2.28867	118	3.55E-06
C2 - CGP	CROONQUIST_IL6_DEPRIVATION_DN	-0.65509	-2.38458	92	1.04E-05
C2 - CGP	CROONQUIST_NRAS_SIGNALING_DN	-0.67335	-2.28670	70	0.00017
C2 - CGP	ISHIDA_E2F_TARGETS	-0.73204	-2.39640	51	0.00020
C2 - CGP	KONG_E2F3_TARGETS	-0.63666	-2.28583	87	0.00037
C2 - CGP	FISCHER_G2_M_CELL_CYCLE	-0.46953	-1.85803	219	0.00060
C2 - CGP	WINZEN_DEGRADED_VIA_KHSRP	0.77066	2.20231	51	0.00153
C2 - CGP	LEE_EARLY_T_LYMPHOCYTE_UP	-0.58703	-2.11496	90	0.01371
C2 - CGP	ZHOU_CELL_CYCLE_GENES _IN_IR_RESPONSE_24HR	-0.54033	-1.99311	112	0.02374
C2 - CGP	GRAHAM_CML_DIVIDING _VS_NORMAL_QUIESCENT_UP	-0.49131	-1.89057	154	0.02503
C2 - CGP	ZWANG_CLASS_3_TRANSIENTLY _INDUCED_BY_EGF	0.55568	1.88056	170	0.03355
C2 - CGP	DEBOSSCHER_NFKB_TARGETS _REPRESSED_BY_GLUCOCORTICOIDS	0.91888	1.98844	13	0.04135
C2 - CGP	SATO_SILENCED_BY_METHYLATION _IN_PANCREATIC_CANCER_1	0.51537	1.78870	229	0.04306
C2 - CGP	MISSIAGLIA_REGULATED _BY_METHYLATION_DN	-0.52915	-1.95187	112	0.04476
C2 - CGP	ZHOU_CELL_CYCLE_GENES _IN_IR_RESPONSE_6HR	-0.58636	-2.05484	78	0.04571
C2 - CGP	KANG_DOXORUBICIN_RESISTANCE_UP	-0.66676	-2.18074	52	0.04658
C2 - CGP	BURTON_ADIPOGENESIS_3	-0.57052	-2.05570	89	0.04673
C2 - CP	NABA_SECRETED_FACTORS	0.77841	2.48568	100	1.06E-10
C2 - CP	NABA_MATRISOME_ASSOCIATED	0.62021	2.13729	215	3.82E-07
C2 - CP:BIOCARTA	BIOCARTA_NKT_PATHWAY	0.88935	2.18814	24	0.00028
C2 - CP:KEGG	KEGG_CYTOKINE_CYTOKINE _RECEPTOR_INTERACTION	0.72398	2.39658	137	2.82E-10
C2 - CP:KEGG	KEGG_JAK_STAT_SIGNALING_PATHWAY	0.73344	2.31259	93	1.43E-06
C2 - CP:KEGG	KEGG_T_CELL_RECEPTOR _SIGNALING_PATHWAY	0.66750	2.13309	101	0.00099
C2 - CP:PID	PID_TCR_CALCIUM_PATHWAY	0.85139	2.16362	28	0.00943
C2 - CP:REACTOME	REACTOME_INTERLEUKIN_10_SIGNALING	0.86284	2.26698	32	0.00034
C2 - CP:REACTOME	REACTOME_INTERLEUKIN _2_FAMILY_SIGNALING	0.78469	2.10566	37	0.03392

C7 - IMMUNESIGDB	GSE41176_UNSTIM_VS_ANTI_IGM_STIM_BCELL_3H_UP	0.66157	2.19835	144	6.29E-06
C7 - IMMUNESIGDB	GSE36891_POLYIC_TLR3_VS_PAM_TLR2_STIM_PERITONEAL_MACROPHAGE_UP	0.68899	2.21034	106	0.00010
C7 - IMMUNESIGDB	GSE30971_CTRL_VS_LPS_STIM_MACROPHAGE_WBP7_HET_2H_UP	0.62221	2.07054	149	0.00012
C7 - IMMUNESIGDB	GSE2770_IL12_AND_TGFB_ACT_VS_ACT_CD4_TCELL_6H_UP	-0.54429	-2.09871	156	0.00013
C7 - IMMUNESIGDB	GSE19923_WT_VS_HEB_AND_E2A_KO_DP_THYMOCYTE_DN	0.58741	1.98930	177	0.00036
C7 - IMMUNESIGDB	GSE19941_UNSTIM_VS_LPS_STIM_IL10_KO_NFKBBP50_KO_MACROPHAGE_UP	-0.50438	-1.93724	171	0.00046
C7 - IMMUNESIGDB	GSE9988_ANTI_TREM1_VS_ANTI_TREM1_AND_LPS_MONOCYTE_DN	0.61679	2.05321	142	0.00087
C7 - IMMUNESIGDB	GSE9988_LPS_VS_VEHICLE_TREATED_MONOCYTE_UP	0.60451	2.01164	149	0.00096
C7 - IMMUNESIGDB	GSE9988_LOW_LPS_VS_VEHICLE_TREATED_MONOCYTE_UP	0.60671	2.02090	151	0.00154
C7 - IMMUNESIGDB	GSE39556_CD8A_DC_VS_NK_CELL_MOUSE_3H_POST_POLYIC_INJ_UP	-0.47574	-1.84481	186	0.00354
C7 - IMMUNESIGDB	GSE30971_WBP7_HET_VS_KO_MACROPHAGE_2H_LPS_STIM_DN	0.59558	1.98366	145	0.00904
C7 - IMMUNESIGDB	GSE25088_WT_VS_STAT6_KO_MACROPHAGE_ROSIGLITAZONE_AND_IL4_STIM_DN	0.56809	1.92144	168	0.00933
C7 - IMMUNESIGDB	GOLDRATH_NAIVE_VS_MEMORY_CD8_TCELL_DN	0.55430	1.87718	177	0.01582
C7 - IMMUNESIGDB	GSE36888_UNTREATED_VS_IL2_TREATED_STAT5_AB_KNOCKIN_TCELL_2H_UP	0.57547	1.92608	157	0.01772
C7 - IMMUNESIGDB	GSE9988_ANTI_TREM1_AND_LPS_VS_VEHICLE_TREATED_MONOCYTES_UP	0.59011	1.96543	145	0.01793
C7 - IMMUNESIGDB	GSE6259_33D1_POS_VS_DEC205_POS_FLT3L_INDUCED_SPLENIC_DC_UP	0.59685	1.96627	131	0.01987
C7 - IMMUNESIGDB	GSE21360_SECONDARY_VS_QUATERNARY_MEMORY_CD8_TCELL_UP	0.59792	1.98086	138	0.02499
C7 - IMMUNESIGDB	GSE15750_DAY6_VS_DAY10_EFF_CD8_TCELL_UP	-0.45610	-1.77962	187	0.02865
C7 - IMMUNESIGDB	GSE36888_UNTREATED_VS_IL2_TREATED_TCELL_17H_DN	0.57885	1.93778	156	0.03133
C7 - IMMUNESIGDB	GSE7852_LN_VS_FAT_TCONV_DN	0.55079	1.86433	171	0.03725
C7 - IMMUNESIGDB	GSE19923_WT_VS_HEB_KO_DP_THYMOCYTE_UP	0.59806	1.96137	123	0.03894
C7 - IMMUNESIGDB	GSE30971_CTRL_VS_LPS_STIM_MACROPHAGE_WBP7_KO_2H_UP	0.58429	1.94690	143	0.04628
C7 - IMMUNESIGDB	GSE42088_UNINF_VS_LEISHMANIA_INF_DC_2H_UP	0.55381	1.87266	169	0.04725

[a]ES, enrichment score; NES, normalized enrichment score (normalized to mean enrichment of random samples of the same size); Adj*P*, Bonferroni-adjusted *P* value; CP, canonical pathways; CGP, chemical and genetic perturbations; PID, Pathway Interaction Database.

Table S9. GWAS enriched diseases for differentially expressed genes in Treg cells of early pregnancy failure subjects, Related to Figure 6.

Term	Overlap	Adj*P*[a]	Odds Ratio	Combined Score	Genes
Celiac disease	9/130	0.01038	6.49421	69.43449	*IL21, RGS1, TNFSF4, PLEK, NOG, THEMIS, IGF2, CTLA4, UBASH3A*
Type 1 diabetes	6/91	0.01559	7.22540	66.81131	*IL21, IL10, POMC, GLIS3, CTLA4, LMO7, UBASH3A*
Itch intensity from mosquito bite	6/64	0.01559	8.94192	82.15101	*IL21, IL10, CLIC5, IFNG, ADAM12, SOX13*

[a]Adj*P*, Bonferroni-adjusted *P* value

Table S10. Antibody reagents used in flow cytometry analysis, Related to Figures 1-5.

Marker	Fluorochrome	Clone	µl / test	Antibody cocktail	Supplier
CCR7	BUV395	3D12	2.5	pre-label	BD Biosciences
CD3	APC-H7	SK7	2	cell-surface	BD Biosciences
CD4	BUV496	SK3	2	cell-surface	BD Biosciences
CD8	BUV737	SK1	0.03	cell-surface	BD Biosciences
CD25	BV786	2-A3	2	cell-surface	BD Biosciences
CD127	PE-Cy7	HIL-7R-M21	2	cell-surface	BD Biosciences
CD45RA	BB515	HI100	2	cell-surface	BD Biosciences
HLADR	BV510	G46-6	2	cell-surface	BD Biosciences
FOXP3	PE-CF594	236A/E7	2	intra-cellular	BD Biosciences
Helios	AlexaFluor 647	22F6	2	intra-cellular	BD Biosciences
CTLA4	PE-Cy5	BNI3	3.5	intra-cellular	BD Biosciences
Ki67	APC-R700	SolA15	2	intra-cellular	eBiosciences
RORγt	PE	Q21-559	2	intra-cellular	BD Biosciences
Tbet	BV421	O4-46	2	intra-cellular	BD Biosciences
